# Bifunctional Hydrogen Bond Donor‐Catalyzed Diels–Alder Reactions: Origin of Stereoselectivity and Rate Enhancement

**DOI:** 10.1002/chem.202004496

**Published:** 2021-01-12

**Authors:** Pascal Vermeeren, Trevor A. Hamlin, F. Matthias Bickelhaupt, Israel Fernández

**Affiliations:** ^1^ Department of Theoretical Chemistry Amsterdam Institute of, Molecular and Life Sciences (AIMMS) Amsterdam Center for, Multiscale Modeling (ACMM) Vrije Universiteit Amsterdam De Boelelaan 1083 1081 HV Amsterdam The Netherlands; ^2^ Institute for Molecules and Materials (IMM) Radboud University Heyendaalseweg 135 6525 AJ Nijmegen The Netherlands; ^3^ Departamento de Química Orgánica I Centro de Innovación, en Química Avanzada (ORFEO-CINQA) Facultad de Ciencias Químicas Universidad Complutense de Madrid 28040 Madrid Spain

**Keywords:** (thio)urea, activation strain model, density functional calculations, Diels–Alder reaction, hydrogen bond donor catalysis, Pauli repulsion

## Abstract

The selectivity and rate enhancement of bifunctional hydrogen bond donor‐catalyzed Diels–Alder reactions between cyclopentadiene and acrolein were quantum chemically studied using density functional theory in combination with coupled‐cluster theory. (Thio)ureas render the studied Diels–Alder cycloaddition reactions *exo* selective and induce a significant acceleration of this process by lowering the reaction barrier by up to 7 kcal mol^−1^. Our activation strain and Kohn–Sham molecular orbital analyses uncover that these organocatalysts enhance the Diels–Alder reactivity by reducing the Pauli repulsion between the closed‐shell filled π‐orbitals of the diene and dienophile, by polarizing the π‐orbitals away from the reactive center and not by making the orbital interactions between the reactants stronger. In addition, we establish that the unprecedented *exo* selectivity of the hydrogen bond donor‐catalyzed Diels–Alder reactions is directly related to the larger degree of asynchronicity along this reaction pathway, which is manifested in a relief of destabilizing activation strain and Pauli repulsion.

## Graphical abstract

**Speed it up**! Quantum chemical activation strain analyses reveal that (thio)urea‐derived bifunctional hydrogen bond organocatalysts accelerate the Diels–Alder reaction between cyclopentadiene and acrolein by reducing the two‐center four‐electron (steric) Pauli repulsion between the occupied π‐orbitals of the reactants and not by enhancing the donor–acceptor orbital interactions, as is traditionally considered.

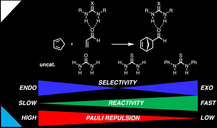

## Introduction

The Diels–Alder (DA) cycloaddition reaction is arguably one of the most useful processes in chemistry due to its ability to produce six‐membered rings in a single reaction step generating up to four stereocenters.^[1]^ Due to the relevance of this transformation in synthesis,^[2]^ an impressive number of organocatalysts have been developed not only to accelerate but also to produce highly enantioselective cycloadditions. In this regard, a wide variety of chiral amines, heterocyclic carbenes, guanidines, ureas, amidinium ions, diols, squaramides, and other Lewis acids have successfully been used to afford the corresponding cycloadduct with high enantiomeric excess.[^3^, ^4^]

**Scheme 1:**
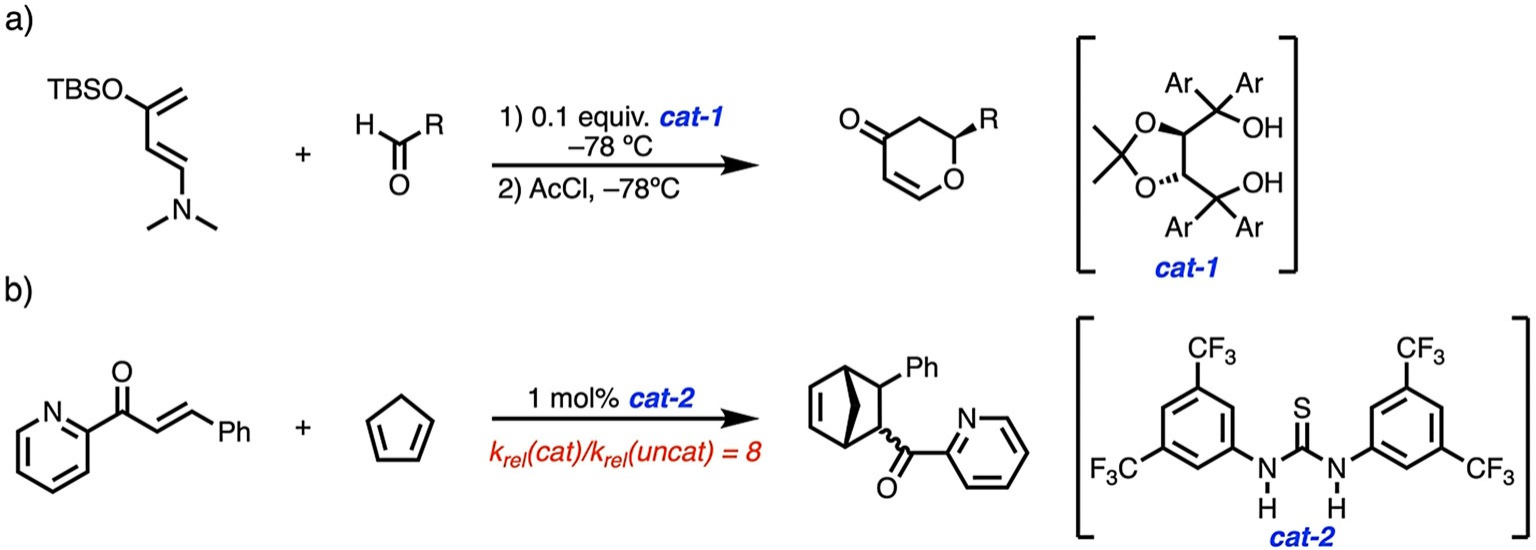
Representative organocatalyzed Diels–Alder cycloaddition reactions involving a) TADDOL and b) Schreiner's thiourea. AcCl = acetyl chloride.

In particular, bifunctional hydrogen bond donor organocatalysts, i.e., molecules able to act as Lewis acid via two hydrogen bonds, have attracted considerable attention in this line of research.^[4]^ For instance, the seminal works by Rawal and co‐workers^[5]^ and Schreiner and co‐workers^[6]^ on the use of TADDOL and thioureas, respectively, as organocatalysts for Diels–Alder reactions should be especially highlighted (Scheme 1). The catalytic activity of these species is generally ascribed to the bidentate nature of the organocatalyst–substrate binding, which favorably preorganizes and activates the substrate.[^3b^, ^6^, ^7^] In analogy with conventional Lewis acid catalysts, this type of activation is widely accepted to be the result of the *lowering of the LUMO of the dienophile* upon binding with the double hydrogen donor catalyst.^[6]^ However, we recently demonstrated that orbital interactions are not the origin of conventional Lewis acid catalysis in Diels–Alder reactions, but, in contrast, a significant reduction of the steric (Pauli) repulsion between the occupied π‐molecular orbitals of the diene and dienophile enhances the Diels–Alder reactivity.^[8]^ It would not be surprising if this *Pauli‐repulsion lowering* concept, which is also operative in iminium‐catalyzed Diels–Alder reactions^[9]^ and dihalogen‐catalyzed aza‐Michael additions,^[10]^ would be the actual driving force behind the catalysis mediated by bifunctional hydrogen bond donor species.

**Scheme 2:**
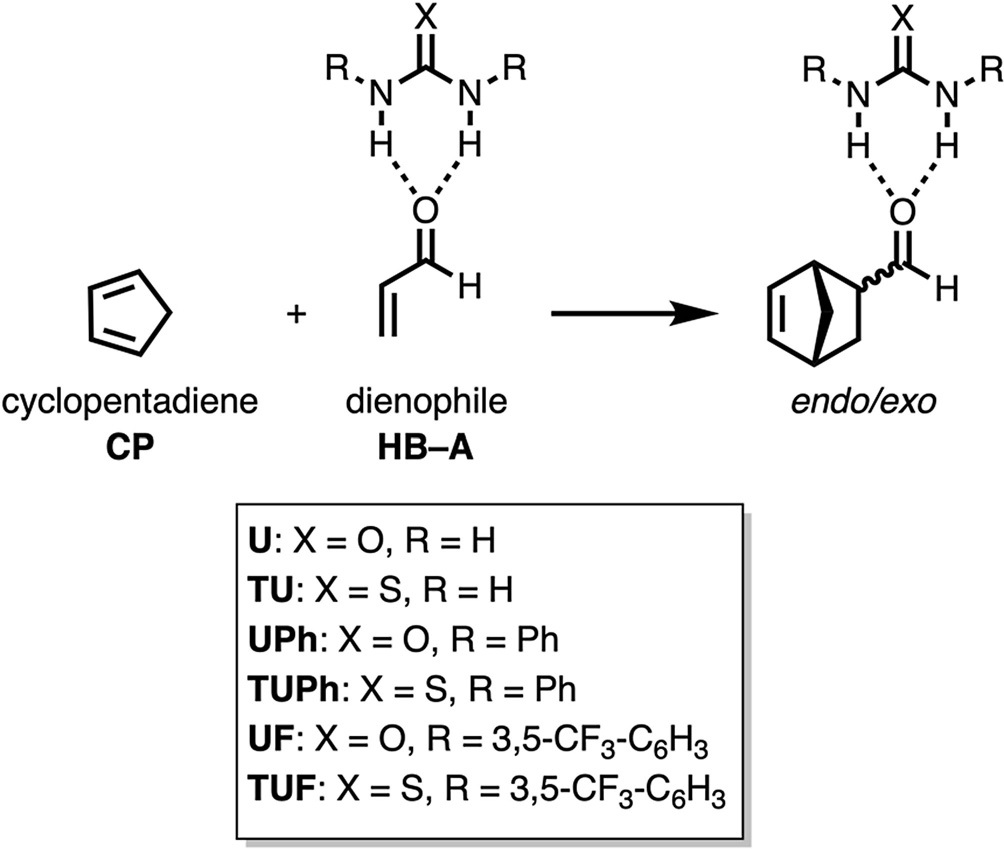
(Thio)urea‐catalyzed Diels–Alder cycloaddition reactions considered in this study.

To check our hypothesis and to gain detailed insight into the hitherto poorly understood mode of activation of bifunctional hydrogen bond donor (HB) species, we selected the Diels–Alder cycloaddition reaction between cyclopentadiene (**CP**) and acrolein (**A**) catalyzed by different, as Lewis acid acting, (thio)urea species analogous to the processes described by Schreiner and co‐workers (Scheme 2).^[6a]^ The *endo*/*exo* selectivity as well as the origin of the rate enhancement are quantitatively explored by means of state‐of‐the‐art computational methods, namely, the activation strain model (ASM) of reactivity^[11]^ in conjunction with quantitative Kohn–Sham molecular orbital theory (KS‐MO) and a matching energy decomposition analysis (EDA).^[12]^ This computational approach has been chosen due to its good performance to understand not only fundamental processes in organic and organometallic chemistry^[11]^ but, in particular, the mode of activation and catalysis in related transformations.[^8^, ^9^, ^10^]

## Computational Methods

Full geometry optimization of all stationary structures and vibrational analyses were carried out at the M06‐2X/def2‐SVPP level[^13^, ^14^] using the Gaussian 16 program.^[15]^ The potential energy surfaces of the studied Diels–Alder reactions were obtained by performing intrinsic reaction coordinate (IRC) calculations.^[16]^ The activation strain model (ASM) and energy decomposition analyses (EDA) were carried out by using the PyFrag 2019^[17]^ and ADF.2018.104^[18]^ programs using the same functional in conjunction with doubly polarized triple‐ζ quality TZ2P basis set^[19]^ on the geometries optimized at M06‐2X/def2‐SVPP. The zeroth‐order regular approximation (ZORA) was used to account for scalar relativistic effects.^[20]^ This level is referred to as ZORA‐M06‐2X/TZ2P//M06‐2X/def2‐SVPP and has been selected because it has been proven to provide accurate activation barriers in related reactions^[8a–^, ^10]^ and is well suited to capture the non‐covalent interactions relevant to reaction kinetics.[^21^, ^22^] Domain‐based local pair natural orbital coupled‐cluster (DLPNO‐CCSD(T)) calculations, with default normalPNO settings, were performed using Orca 4.0.1^[23]^ using the def2‐QZVPP^[14]^ basis set on the M06‐2X/def2‐SVPP optimized geometries.

## Results and Discussion

**Figure 1:**
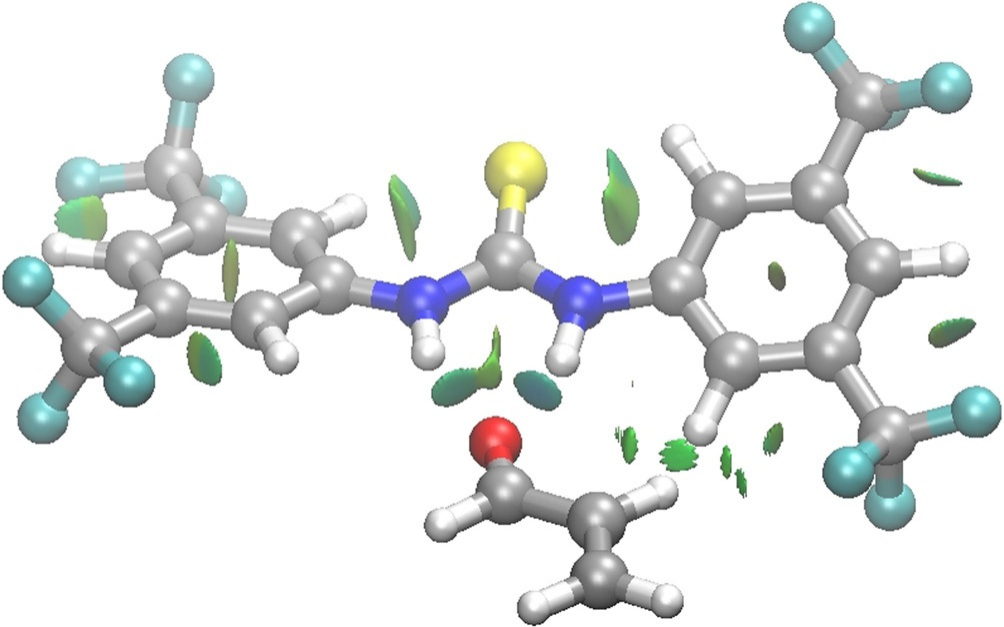
Contour plots of the reduced density gradient isosurfaces (density cutoff = 0.03 au) for the **TUF‐A** complex. The green surfaces indicate attractive non‐covalent interactions.

We first analyzed the nature and strength of the (thio)urea‐acrolein (**HB‐A**) interaction, which is crucial to understand the catalysis mediated by these bifunctional organocatalysts. In all cases, the (thio)urea hydrogen bond donor (HB) catalysts form a bidentate complex via a bifurcated hydrogen bond to **A**. This typical stabilizing double hydrogen bond interaction can be easily visualized by means of the NCIPLOT method.^[24]^ As shown in Figure 1, for the complex involving Schreiner's thiourea **TUF**,^[6]^ there exist two clear non‐covalent attractive interactions (green surfaces) between both N−H hydrogen bond donors of **TUF** and the carbonyl oxygen atom of **A** acting as a hydrogen bond acceptor, which confirms the occurrence of both hydrogen bonds. In this particular complex, and in agreement with previous experimental findings,^[25]^ there are two additional stabilizing C−H⋅⋅⋅S interactions between the Lewis basic sulfur atom and the *ortho* hydrogen atoms of the aryl groups as a consequence of the polarization exerted by the electron‐withdrawing CF_3_ substituents on the aryl groups. The latter non‐covalent interactions are suggested to hinder the rotation of the aryl groups.^[6a]^

More detailed quantitative insight into the (thio)urea‐acrolein (**HB‐A**) complexation can be obtained by using the energy decomposition analysis (EDA)^[12]^ Scheme at the ZORA‐M06‐2X/TZ2P//M06‐2X/def2‐SVPP level. This method decomposes the interaction energy (Δ*E*_int_) between HB and **A** into the following three chemically meaningful energy terms: classical electrostatic interaction (Δ*V*_elstat_), Pauli repulsion (Δ*E*_Pauli_) between closed‐shell orbitals which is responsible for steric repulsion, and stabilizing orbital attractions (Δ*E*_oi_) that account, among others, for HOMO–LUMO interactions. As gathered in Table 1, the interaction between the different HBs and **A**, derived from the double hydrogen bond, ranges from −7 to −14 kcal mol^−1^, which, not surprisingly, is significantly weaker than the value computed for the analogous interaction involving strong Lewis acids such as BF_3_ or AlCl_3_ but comparable to those species involving weaker Lewis acids such as SnCl_4_ or TiCl_4_.^[8a]^ As shown in Table 1, the Δ*E*_int_ becomes increasingly more stabilizing from **U < UPh** < **UF** and **TU** < **TUPh** < **TUF**, which, interestingly, follows the same trend as their experimentally determined acidities^[26]^ and the observed acceleration of the Diels–Alder cycloaddition involving the analogous methyl vinyl ketone (**TU** < **TUPh** < **TUF**).^[6a]^ The corresponding bifurcated hydrogen bond length between H^1^⋅⋅⋅O=C and H^2^⋅⋅⋅O=C becomes steadily shorter along this series, in line with the increasing bond strength. One exception, however, is **TUPh**, which has a slightly longer hydrogen bond length than **UPh**. As expected, the interaction term is dominated by the Δ*V*_elstat_ term, confirming the electrostatic nature of this hydrogen bonding interaction. Although the Δ*V*_elstat_ term is more than twice as strong, the Δ*E*_oi_ is not negligible and also follows the same trend as Δ*E*_int_ and Δ*V*_elstat_. As a result, the LUMO of the complex, i.e., the π*_C=C_ molecular orbital located on the C=C double bond of **A**, becomes more stabilized from **U** to **TUF**, which is consistent with the widely accepted LUMO lowering concept (vide supra).^[6]^ The donor–acceptor interaction between HB and **A**, which transfers charges from **A** to HB, results in a slightly positively charged **A**. This positive field, in turn, lowers (i.e., stabilizes) the LUMO on **A** and this effect becomes more prominent as the donor‐acceptor interaction between HB and **A** is strengthened. Furthermore, it becomes visible that thiourea‐based HBs have, in line with their experimentally determined stronger acidity (p*K*_A_ = 21.1 and 26.9, for thiourea and urea, respectively),[^26^, ^27^] a consistently more stabilizing interaction with **A** than their urea counterparts, which suggests that thiourea species are better catalysts than their urea analogues.

**Table 1:** Energy decomposition analysis terms (in kcal mol^−1^), LUMO (π*_C=C_) energy (in eV), and H⋅⋅⋅O=C distances (in Å), computed on hydrogen bond donor–acrolein (**HB‐A**) complexes.^[a]^

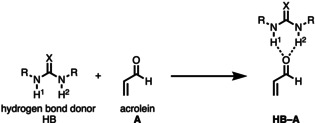							
HB	Δ*E*_int_	Δ*V*_elstat_	Δ*E*_Pauli_	Δ*E*_oi_	*ϵ*_LUMO_	*r*(H^1^⋅⋅⋅O=C)	*r*(H^2^⋅⋅⋅O=C)
U	−7.1	−9.3	5.5	−3.2	−1.7	2.147	2.144
TU	−8.5	−11.0	6.6	−4.1	−1.8	2.115	2.101
UPh	−9.1	−12.0	7.6	−4.7	−1.9	2.053	2.052
TUPh	−10.4	−13.9	9.5	−6.1	−1.7	2.078	2.067
UF	−13.0	−15.3	9.0	−6.8	−2.3	2.084	1.955
TUF	−13.8	−16.3	10.2	−7.7	−2.3	2.076	1.935

[a] The hydrogen bond donor (HB) and acrolein (**A**) constitute the two interacting fragments. All data computed at the ZORA‐M06‐2X/TZ2P//M06‐2X/def2‐SVPP level.

After analyzing the bonding situation in the **HB‐A** complexes, we focused on the Diels–Alder reaction of these activated species with cyclopentadiene. The electronic reaction barriers (Δ*E* ^≠^), reaction energies (Δ*E*_rxn_), and HOMO_**CP**_–LUMO_**HB‐A**_ orbital energy gaps (Δ*ϵ*_H–L_) of the uncatalyzed and HB‐catalyzed Diels–Alder (DA) reaction between cyclopentadiene (**CP**) and HB‐acrolein complexes (**HB‐A**) (Scheme 2) are provided in Table 2. In agreement with previous calculations,^[6b]^ all studied reactions occur in a concerted asynchronous manner through the corresponding six‐membered transition state (see Figure S1 for the optimized transition state structures).

**Table 2:** Electronic reaction barriers (Δ*E* ^≠^), reaction energies (Δ*E*_rxn_) (in kcal mol^−1^), and HOMO_**CP**_–LUMO_**HB‐A**_ energy gaps (Δ*ϵ*_H–L_) (in eV) for the uncatalyzed and hydrogen bond donor‐catalyzed Diels–Alder reaction between cyclopentadiene and acrolein.

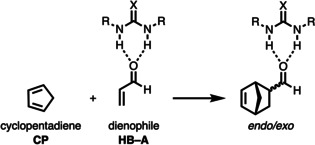					
HB‐A	Cycloadduct	Δ*E* ^≠[a]^	Δ*E* ^≠[b]^	Δ*E*_rxn_^[a]^	Δ*ϵ*_H–L_^[a]^
**A**	*endo*	13.1	14.3	−25.3	−6.6
	*exo*	13.6	14.8	−25.1	−6.6
**U‐A**	*endo*	9.3	11.1	−25.8	−5.9
	*exo*	8.9	10.4	−25.5	−5.9
**TU‐A**	*endo*	8.2	9.5	−25.9	−5.7
	*exo*	8.3	10.0	−25.5	−5.7
**UPh‐A**	*endo*	8.4	8.2	−27.4	−5.6
	*exo*	6.4	6.4	−29.0	−5.6
**TUPh‐A**	*endo*	7.5	8.9	−27.0	−5.8
	*exo*	6.4	8.0	−28.2	−5.8
**UF‐A**	*endo*	7.2	7.2	−27.0	−5.3
	*exo*	6.7	6.0	−26.8	−5.3
**TUF‐A**	*endo*	7.0	7.8	−26.9	−5.3
	*exo*	6.2	7.0	−27.2	−5.3

[a] Computed at the ZORA‐M06‐2X/TZ2P//M06‐2X/def2‐SVPP level. [b] Computed at the DLPNO‐CCSD(T)/def2‐QZVPP//M06‐2X/def2‐SVPP level.

According to the computed ZORA‐M06‐2X/TZ2P//M06‐2X/def2‐SVPP barrier energies, which agree well with the more accurate DLPNO‐CCSD(T)/def2‐QZVPP//M06‐2X/def2‐SVPP values, three distinct trends can be observed. In the first place, we, surprisingly, found that the *endo* selectivity of the widely studied uncatalyzed DA reaction between **CP** and **A** changes to *exo* selectivity when this reaction is catalyzed by a HB. The reaction barriers of the *exo* HB‐catalyzed DA reaction pathways are 0.4 to 2.1 kcal mol^−1^ lower in energy than the corresponding *endo* pathways. There is, however, one exception, namely, the Diels–Alder reaction between **CP** and **TU‐A**, where the *exo* and *endo* reaction barriers are nearly identical. Secondly, the Diels–Alder reactions catalyzed by thiourea‐based HBs proceed with a lower reaction barrier than those promoted by their urea‐based analogues, which confirms the superior catalytic activity of thioureas. Their reaction energies are, on the other hand, nearly identical. Thirdly, introducing a HB catalyst significantly accelerated the DA lowering the reaction barrier 4–7 kcal mol^−1^. This rate enhancement becomes more significant when the hydrogen atom of the parent (thio)urea (**U‐A**, **TU‐A**) is replaced by a phenyl group (**UPh‐A**, **TUPh‐A**) and even more pronounced, when the aryl groups bear the strong electron‐withdrawing CF_3_ groups (**UF‐A**, **TUF‐A**), which, as commented above, follows the same trend as the computed Δ*E*_int_ of the **HB‐A** complexes and is consistent with the experimental findings.^[6a]^ Furthermore, there is a modest linear correlation (R^2^ = 0.88 for *endo*; R^2^ = 0.83 for *exo*) between the computed reaction barriers (Δ*E* ^≠^) and the corresponding HOMO_**CP**_–LUMO_**HB‐A**_ orbital energy gaps (Δ*ϵ*_H–L_, see Figure S2). This suggests that the reduced reaction barrier, upon coordination of a catalyzing HB to **A**, might be related to the lowering of the LUMO_**HB‐A**_. We will show later that, similar to our previous study on Lewis acid‐catalyzed Diels–Alder reactions,^[8]^ this is not the case for these hydrogen bond donor‐catalyzed Diels–Alder reactions.

### *endo*/*exo* Stereoselectivity

**Figure 2:**
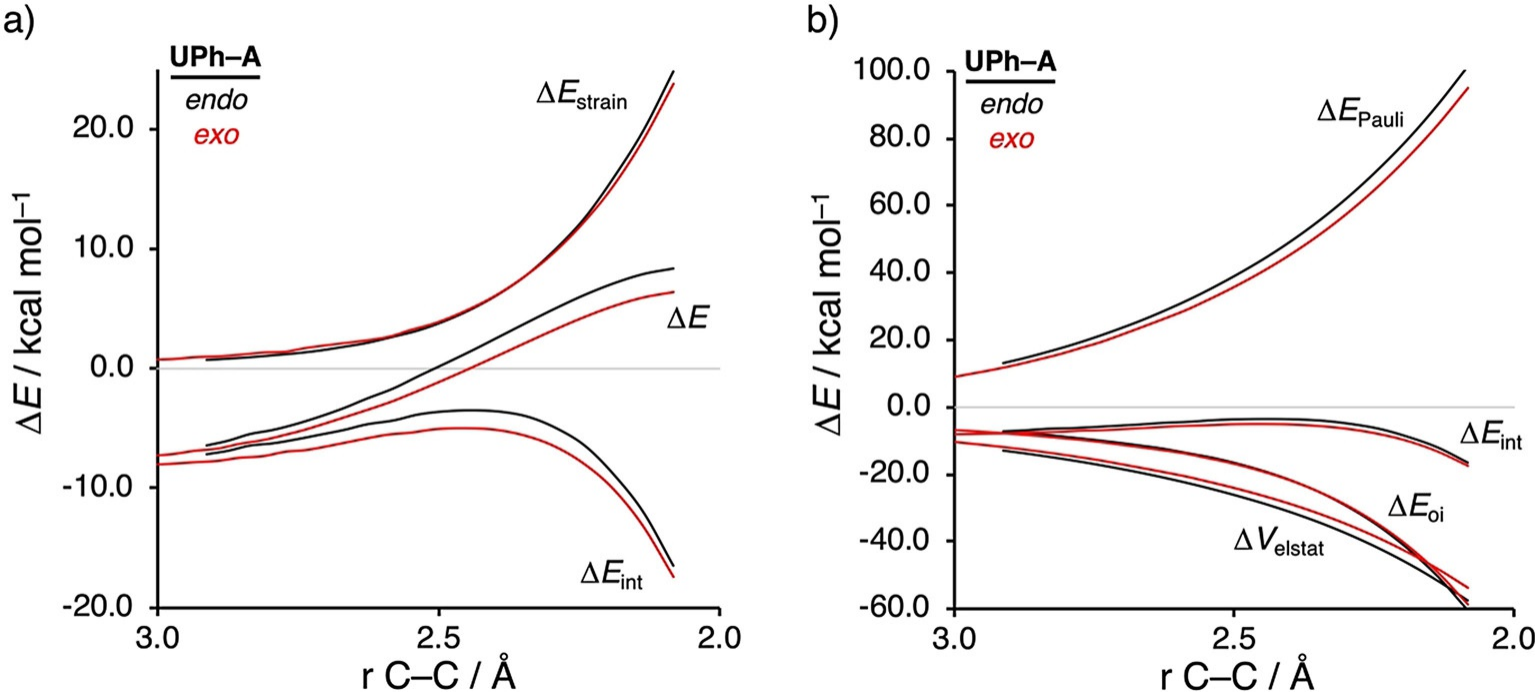
a) Activation strain analyses and b) energy decomposition analyses of the catalyzed *endo* (black lines) and *exo* (red lines) Diels–Alder reactions between **CP** and **UPh‐A** complex, where the energy values are projected onto the shorter newly forming C_**CP**_⋅⋅⋅C_β_ bond between **CP** and the **UPh‐A**, computed at the ZORA‐M06‐2X/TZ2P//M06‐2X/def2‐SVPP level.

Before exploring the factors controlling the rate enhancement induced by the organocatalysts, we first aim to understand why coordinating a HB to **A** significantly alters the *endo*/*exo* selectivity by applying the activation strain model (ASM) of reactivity.^[11]^ This method decomposes the electronic energy (Δ*E*) into two terms: the strain (Δ*E*_strain_) that results from the distortion of the individual reactants and the interaction (Δ*E*_int_) between the deformed reactants along the reaction coordinate, defined in this case as the IRC projection onto the shorter newly forming C_**CP**_⋅⋅⋅C_β_ bond.^[28]^ To this end, we have analyzed the *endo*/*exo* selectivity of the process involving **UPh‐A**, which has the largest, and hence clearest, difference between the *endo* and *exo* reaction barriers (see Figure 2). Note that the activation strain diagrams (ASD) of all other reactions can be found in the Supporting Information (Figures S3–S6). The *exo* selectivity of the **CP**+**UPh‐A** cycloaddition is the result of both a less destabilizing activation strain and a more stabilizing interaction energy (Figure 2 a). Surprisingly, the stronger interaction energy computed for the *exo* pathway is not dictated by the electrostatic interaction (which still favors *endo*, just as in the case of the uncatalyzed reaction which does go via *endo*; see Figure S3) but by the reduction in the steric (Pauli) repulsion (Figure 2 b). The less destabilizing strain energy for the *exo* pathway can be ascribed to the larger degree of asynchronicity compared to *endo* (*exo*: Δ*r* ^TS^_C⋅⋅⋅C_ = 0.40 Å, *endo*: Δ*r* ^TS^_C⋅⋅⋅C_ = 0.35 Å, where Δr^TS^_C⋅⋅⋅C_ is the difference between the newly forming C⋅⋅⋅C bond lengths in the TS), which leads to a lower degree of deformation of the reactants since the C_**CP**_⋅⋅⋅C_β_ bond forms ahead of the C_**CP**_⋅⋅⋅C_α_ bond (see Figure S1).

**Figure 3:**
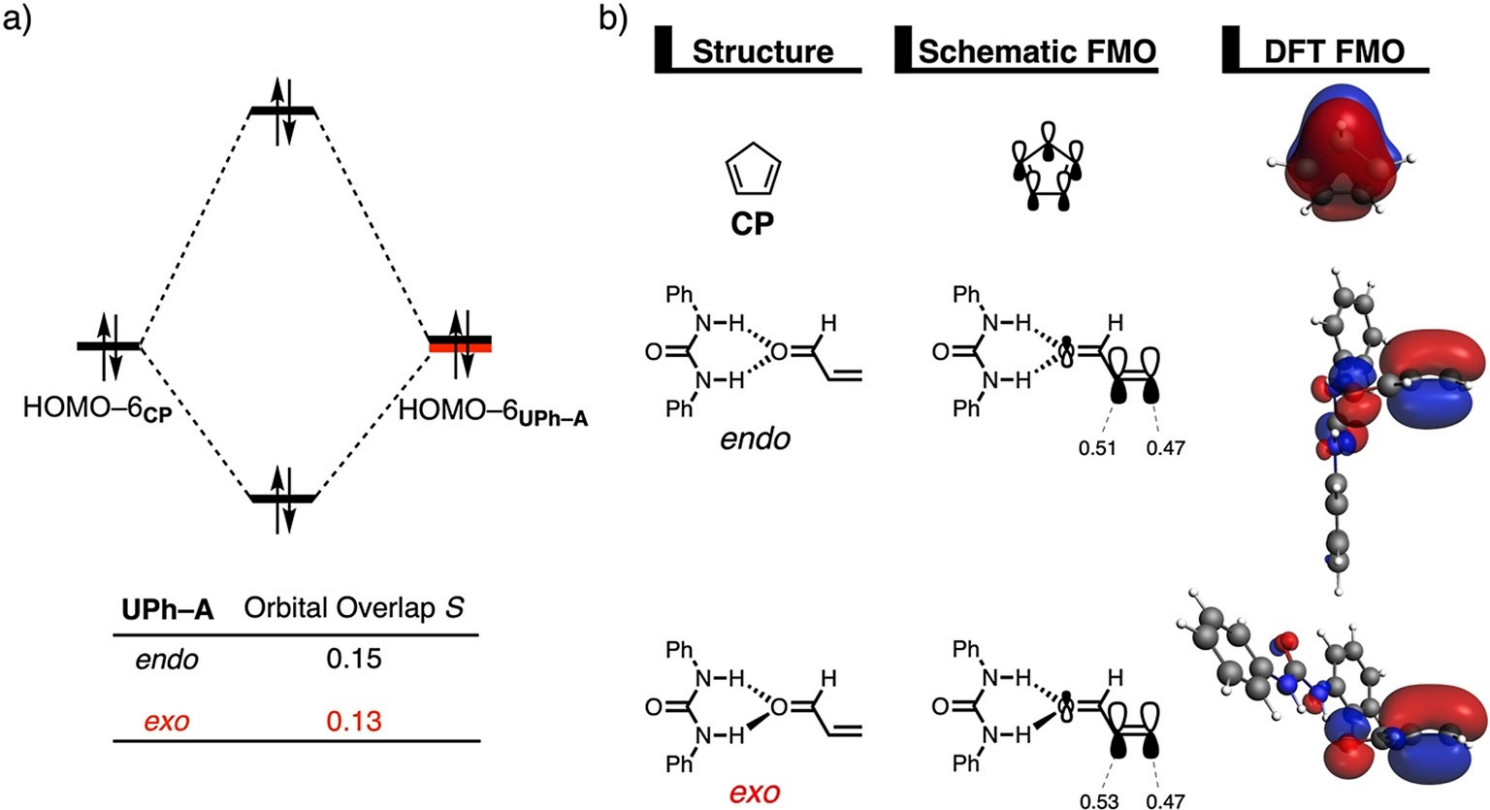
a) Molecular orbital diagram and the most significant occupied orbital overlaps of the *endo* and *exo* Diels–Alder reactions between **CP** and **UPh‐A** and b) key occupied orbitals (isovalue = 0.03 au), where the MO coefficients of the carbon 2*p_z_* atomic orbitals, contributing to the occupied orbitals of the **UPh‐A**, are shown. Computed at the transition state geometries where the C_**CP**_⋅⋅⋅C_β_ bond length between **CP** and the **UPh‐A** is 2.08 Å at the ZORA‐M06‐2X/TZ2P//M06‐2X/def2‐SVPP level.

The origin of the less destabilizing Pauli repulsion for the *exo* Diels–Alder reaction pathway between **CP** and **UPh‐A** complex was further investigated by performing a Kohn–Sham molecular orbital (KS‐MO) analysis.[^12b^, ^29^] The occupied molecular orbitals of **CP** and **UPh‐A**, following both the *endo* and *exo* pathways, were quantified at the transition state geometries where the C_**CP**_⋅⋅⋅C_β_ bond length between **CP** and the **UPh‐A** is 2.08 Å (Figure 3 a). The most important occupied MO of **UPh‐A** involved in the two‐center four‐electron interaction, which determines the underlying differences in Pauli repulsion between the *endo* and *exo* pathway, is the HOMO−6. This particular orbital corresponds to the π‐molecular orbital located on the reactive C=C double bond of **A**. The contributing occupied orbital of **CP** is the HOMO−6 where all 2p_π_ AOs and the σ_C‐H_ (pseudo‐π), located on the reacting C=C double bonds and the methylene bridge, respectively, are in‐phase. Along the *exo* pathway, the orbital overlap between HOMO−6_**CP**_ and HOMO−6_**UPh‐A**_ is smaller (*S =* 0.13) and, therefore, less destabilizing than along the *endo* pathway (*S =* 0.15) (see Figure S7 for the evolution of the orbital overlap along the reaction coordinate). This difference in orbital overlap is a direct consequence of the previously discussed different degrees in asynchronicity, which is, as we have previously shown in our analysis of iminium‐catalyzed Diels Alder reactions,^[9]^ induced by their difference in MO coefficients of the 2*p_z_* atomic orbital on the α‐carbon of the dienophile. The larger this MO coefficients, the larger the degree of asynchronicity. As shown in Figure 3 b, for the *endo* pathway the MO coefficient on the α‐carbon is 0.51, while for the *exo* pathway it is 0.53, resulting in a more destabilizing closed‐shell repulsion at the α‐carbon side for the latter. This effect gets compensated by the elongation of the C_**CP**_⋅⋅⋅C_α_ bond distance along the *exo* pathway, making this reaction more asynchronous. As a result, along the more asynchronous *exo* pathway, the reactants have less orbital overlap at the α‐carbon of the dienophile due to a longer the C_**CP**_⋅⋅⋅C_α_ bond length, manifesting in less destabilizing Pauli repulsion and a lower reaction barrier compared to the *endo* pathway.

One might expect that **CP**, along the *endo* pathway, also has a larger steric (Pauli) repulsion with the HB catalyst. In order to test this hypothesis, we performed a numerical experiment in which we evaluate the interaction between **CP** and **UPh** in the position they obtain in the transition state geometries used for the analysis in Figure 3 (Table S1). Surprisingly, we found a more favorable C−H⋅⋅⋅π interaction between **CP** and the phenyl group of **UPh** for the *exo* than for the *endo* pathway (Δ*E*_int_^*endo*^ = −2.7 kcal mol^−1^; Δ*E*_int_^*exo*^ = −3.3 kcal mol^−1^). As a result of this stronger interaction along the *exo* pathway, the **CP** and **UPh** distance is shorter which, as expected, also goes with a more destabilizing Pauli repulsion compared to the *endo* pathway (Δ*E*_Pauli_^*endo*^ = 1.3 kcal mol^−1^; Δ*E*_Pauli_^*exo*^ = 2.1 kcal mol^−1^). Note that this enhanced interaction for the *exo* pathway between **CP** and HB has also been observed for the other HBs, with an exception for **U** and **TUF** for which the interaction is equal or is slightly stronger along the *endo* pathway. Thus, these findings indicate that besides having less Pauli repulsion between **CP** and **HB‐A** along the *exo* pathway, the more favorable interaction between the **CP** and HB also induce an *exo* selective preference for this reaction.^[30]^

### Transitioning from urea‐ to thiourea‐based hydrogen bond donor catalysts

**Figure 4:**
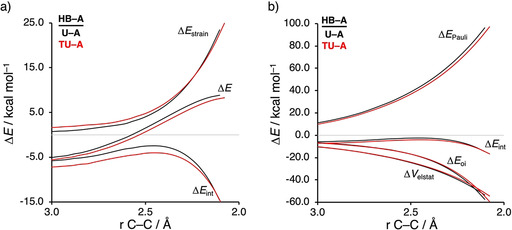
a) Activation strain analyses and b) energy decomposition analyses of the *exo* Diels–Alder reactions between **CP** and **U‐A** (black lines) and **TU‐A** (red lines), where the energy values are projected onto the shorter newly forming C_**CP**_⋅⋅⋅C_β_ bond between **CP** and **HB‐A**, computed at the ZORA‐M06‐2X/TZ2P//M06‐2X/def2‐SVPP level.

Next, we want to establish why thiourea‐based HBs accelerate the Diels–Alder reaction between **CP** and **A** to a larger extent than urea‐based HBs. Figure 4 shows the activation strain diagrams (ASDs) from the reactants to the transition states for the *exo* Diels–Alder reaction between **CP** and **A** catalyzed by the parent urea (**U**) and thiourea (**TU**) HBs. The Diels–Alder reaction following the *endo* pathway, as well as the reactions catalyzed with the larger **UPh‐A** and **TUPh‐A** HBs show the same, albeit less pronounced, features (see Figures S8–S10). The enhanced reactivity of the Diels–Alder reaction involving **TU‐A** originates from both a less destabilizing strain energy (in the transition state region) and a more stabilizing interaction energy compared to the analogous process involving **U‐A** (Figure 4 a). The difference in strain energy can again be ascribed to the different degrees of asynchronicity of these reactions, which is the largest for **TU‐A** (**U‐A**: Δ*r* ^TS^_C⋅⋅⋅C_ = 0.34 Å, **TU‐A**: Δ*r* ^TS^_C⋅⋅⋅C_ = 0.41 Å), leading to less deformation of the reactants up until the transition state. The more stabilizing interaction energy computed for the **TU‐A**+**CP** cycloaddition is, according to the EDA (Figure 4 b), exclusively originating from a less destabilizing Pauli repulsion. The electrostatic and orbital interactions, on the contrary, are less stabilizing for **TU‐A** compared to **U‐A**.

**Figure 5:**
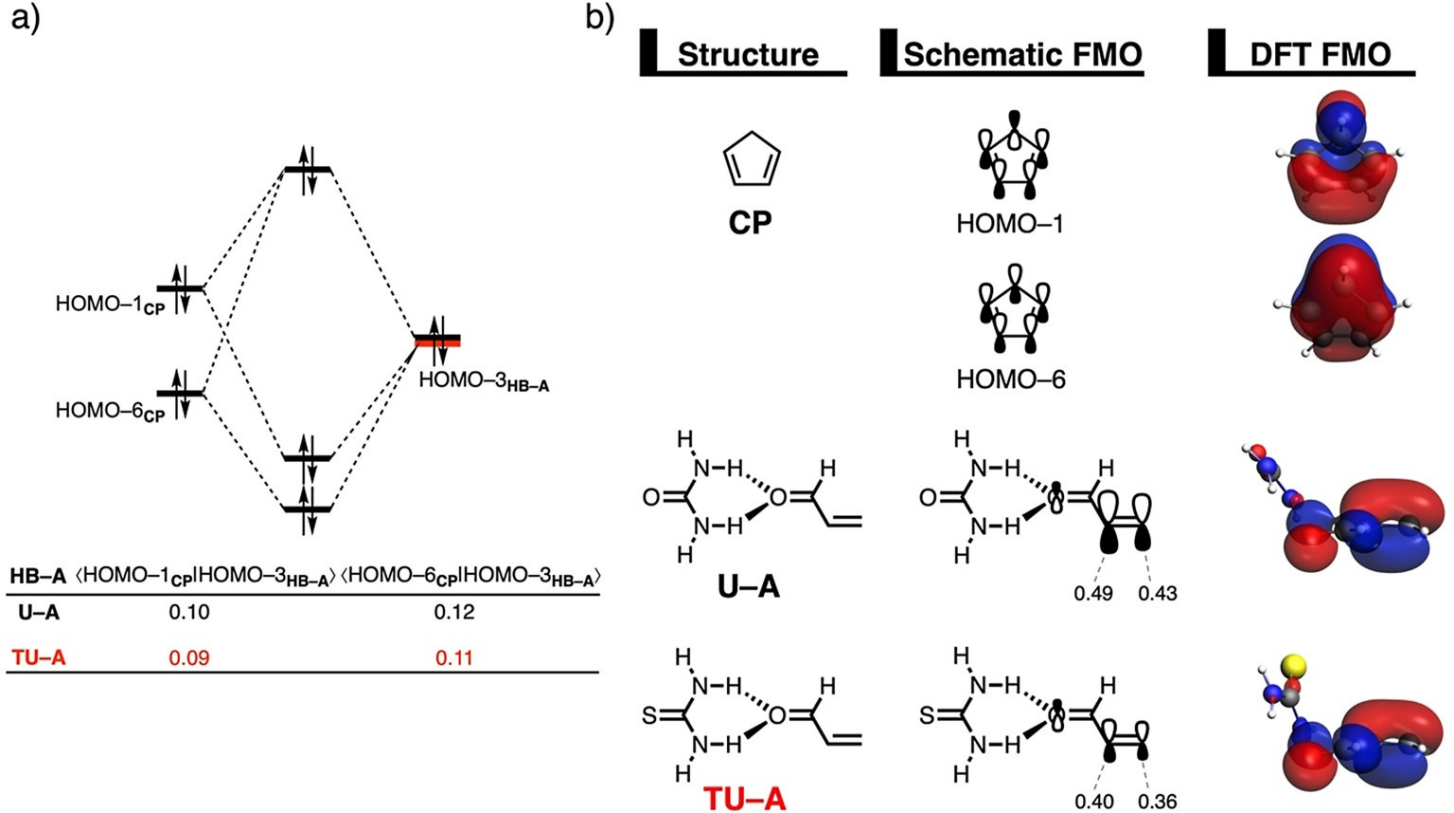
a) Molecular orbital diagram and the most significant occupied orbital overlaps of the *exo* Diels–Alder reactions between **CP** and dienophiles **U‐A** and **TU‐A** and b) key occupied orbitals (isovalue = 0.03 au), where the MO coefficients of the carbon 2*p_z_* atomic orbitals, contributing to the occupied orbitals of **HB‐A** are shown. Computed at consistent geometries with a C_**CP**_⋅⋅⋅C_β_ bond length between **CP** and **HB‐A** of 2.10 Å at the ZORA‐M06‐2X/TZ2P//M06‐2X/def2‐SVPP level.

The less destabilizing Pauli repulsion for the reaction involving **TU‐A** derives from a reduced occupied–occupied orbital overlap with the incoming **CP**. The occupied molecular orbitals of **CP** and the **U‐A** and **TU‐A** complexes were quantified at consistent geometries with a C_**CP**_⋅⋅⋅C_β_ bond length between **CP** and **HB‐A** of 2.10 Å (Figure 5 a).^[31]^ Analysis at a consistent geometry, instead of at the transition state, is advised when the transition states occur at different points on the reaction coordinate. A single‐point analysis on the transition state geometries result in skewed conclusions since the position of the transition state (i.e., early‐ or late‐transition state) has a significant impact on the magnitude of the energy terms.^[11a]^ The occupied π‐MO of **HB‐A** contributing most to the trend in two‐center four‐electron (Pauli) repulsion, is the HOMO−3 of both **U‐A** and **TU‐A**, which corresponds to the π‐molecular orbital predominantly located on the reactive C=C double bond of **A**. Furthermore, **CP** has two π‐MOs that are contributing to the built‐up of Pauli repulsion, namely the HOMO−1 and HOMO−6 where, for the former, the 2p_π_ AOs on the reacting C=C double bonds and the σ_C‐H_ (pseudo‐π) on the methylene bridge are out‐of‐phase whereas, for the latter, they are in‐phase. The computed HOMO–HOMO overlaps decrease from ⟨HOMO−1_**CP**_|HOMO−3_**U‐A**_⟩ = 0.10 and ⟨HOMO−6_**CP**_|HOMO−3_**U‐A**_⟩ = 0.12 for **U‐A** to ⟨HOMO−1_**CP**_|HOMO−3_**TU‐A**_⟩ = 0.09 and ⟨HOMO−6_**CP**_|HOMO−3_**TU‐A**_⟩ = 0.11 for **TU‐A**. The differences in orbital overlap between **U‐A** and **TU‐A** can be attributed to two different phenomena: (i) the thiourea‐based HB of **TU‐A** is, due to its more acidic nature and, therefore, lower‐lying LUMO,^[25]^ able to better polarize the π‐MO of the dienophile away from the reactive C=C double bond of **A** than **U‐A**, and (ii) the DA reaction involving **TU‐A** is more asynchronous than the analogous reaction with **U‐A**. The stronger donor–acceptor interaction between the two σ*_N‐H_ orbitals of the hydrogen bond donor catalyst **TU** and the oxygen lone pair of **A** (see Table 1) results in a charge transfer from **A** to **TU** which is manifested in a smaller orbital amplitude on the C=C double bond of **A** (see MO coefficients in Figure 5 b), compared to the interaction between **U** and **A**. We expect, based on the surmounting evidence provided by our recent work,[^8^, ^9^, ^10^] that the stronger the (Lewis) acid, the greater the catalysis will be. This is due to the relationship between the strength of the (Lewis) acid and the polarization of the orbital density away from the reactive center, which accelerates the reaction via a reduction of activation strain and steric repulsion. Furthermore, in analogy with the analysis of the *exo* preference of **UPh‐A**, the larger degree of asynchronicity for **TU‐A** also contributes to the reduction of destabilizing orbital overlap between the reactants.

### Catalytic effect of hydrogen bond donor catalysts

**Figure 6:**
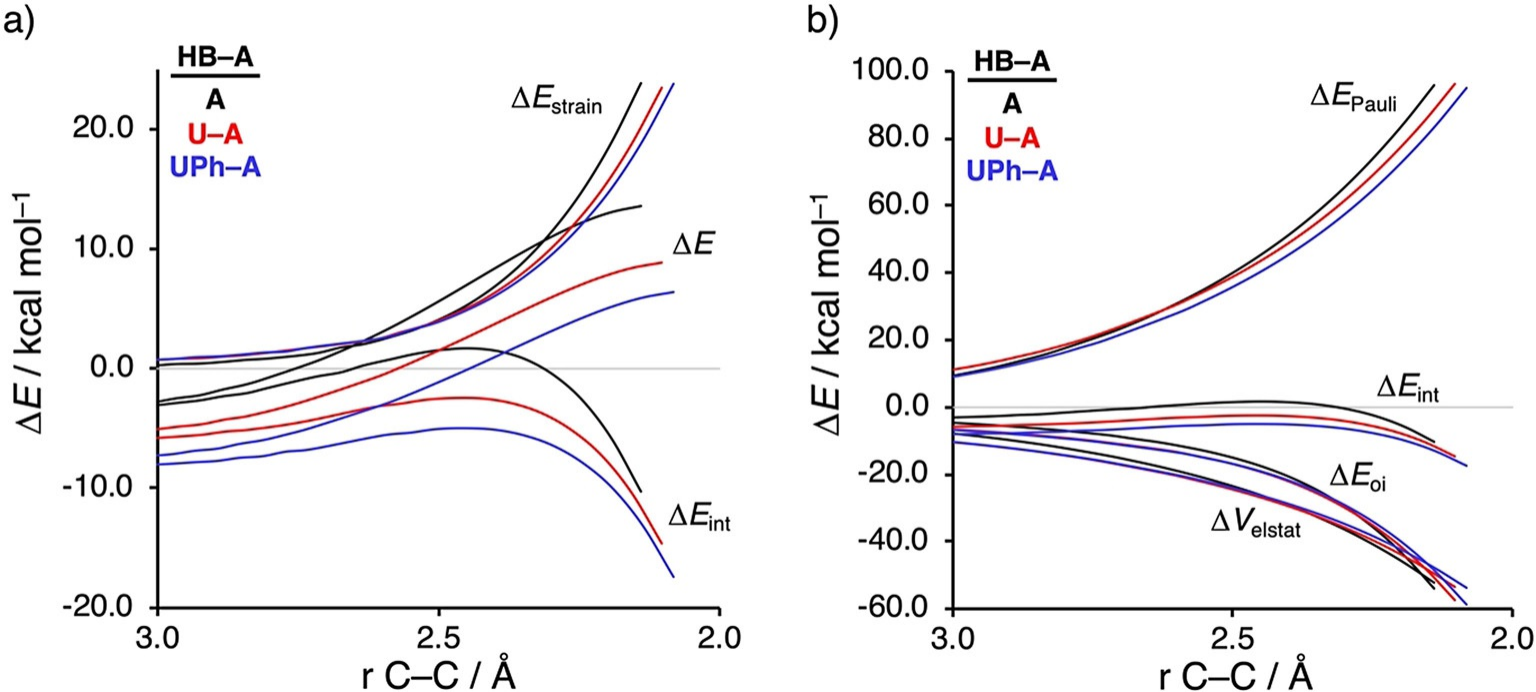
a) Activation strain analyses and b) energy decomposition analyses of the *exo* Diels–Alder reactions between **CP** and **A** (black lines), **U‐A** (red lines), and **UPh‐A** (blue lines), where the energy values are projected onto the shorter newly forming C_**CP**_⋅⋅⋅C_β_ bond between **CP** and **HB‐A**, computed at the ZORA‐M06‐2X/TZ2P//M06‐2X/def2‐SVPP level.

Lastly, we examined the actual catalytic effect of the HBs, in other words, why do HBs accelerate the Diels–Alder reaction and why does this effect become more pronounced when the hydrogen atom of the parent (thio)ureas (**U**/**TU)** are replaced by aryl groups. To this end, we have analyzed and compared the uncatalyzed *exo* DA reaction with the HB‐catalyzed **U‐A** and **UPh‐A** DA reactions (see Figure 6). The accelerated reactivity, i.e., lower reaction barrier, for the HB‐catalyzed compared to the uncatalyzed DA reactions originates from both a less destabilizing strain energy and, to a greater extent, a more stabilizing interaction energy between the deformed reactants along the entire reaction coordinate (Figure 6 a). The *endo* reaction pathway as well as the series catalyzed by thiourea‐based HBs, i.e., **A**, **TU‐A**, **TUPh‐A**, exhibit identical reactivity trends and are provided in the Supporting Information (Figures S11–S13).

The difference in strain energy can again be explained by looking at the degree of asynchronicity, which is the largest for **UPh‐A** (**A**: Δ*r* ^TS^_C⋅⋅⋅C_ = 0.21 Å, **U‐A**: Δ*r* ^TS^_C⋅⋅⋅C_ = 0.34 Å, **UPh‐A**: Δ*r* ^TS^_C⋅⋅⋅C_ = 0.40 Å). The higher degree of asynchronicity of **UPh‐A** leads to a lower degree of deformation of the reactants since the C_**CP**_⋅⋅⋅C_α_ bond forms behind of the C_**CP**_⋅⋅⋅C_β_ bond (see Figure S1). As previously discussed, the differences in degrees of asynchronicity are originating from the asymmetry of the π‐MO located on the reactive C=C double bond of the dienophile, which is induced by the coordination of the HB catalyst (Figure S14). The stronger the HB catalyst coordinates to **A**, the more significant asymmetry in the π‐MO and hence the asynchronicity of the DA reaction becomes.^[9]^ To understand why the interaction energy becomes increasingly more stabilizing from **A** to **U‐A** to **UPh‐A**, we applied the energy decomposition analysis (EDA) (Figure 6 b). Interestingly, we find that the decrease in destabilizing Δ*E*_Pauli_ is the actor behind the more stabilizing Δ*E*_int_ when going from **A** to **U‐A** and to **UPh‐A**. In contrast, the Δ*V*_elstat_ and Δ*E*_oi_ terms are more stabilizing for the uncatalyzed DA reaction, because both terms are weakened by the donor–acceptor interaction between HB and **A**. This donor–acceptor (i.e., charge transfer) interaction results in less negative charge on the reactive C=C double bond and, therefore, a less stabilizing Δ*V*_elstat_ for the HB‐catalyzed reactions and, as we will discuss later, it also leads to a significant reduction in inverse electron demand orbital interaction (i.e. Δ*E*_oi_). This finding confirms our initial hypothesis that, similar to Lewis acid or iminium catalysis,[^8^, ^9^] the *Pauli‐repulsion lowering* rather than the *LUMO lowering* is the actual mechanism behind the bifunctional hydrogen bond donor catalysis in Diels–Alder reactions.

**Figure 7:**
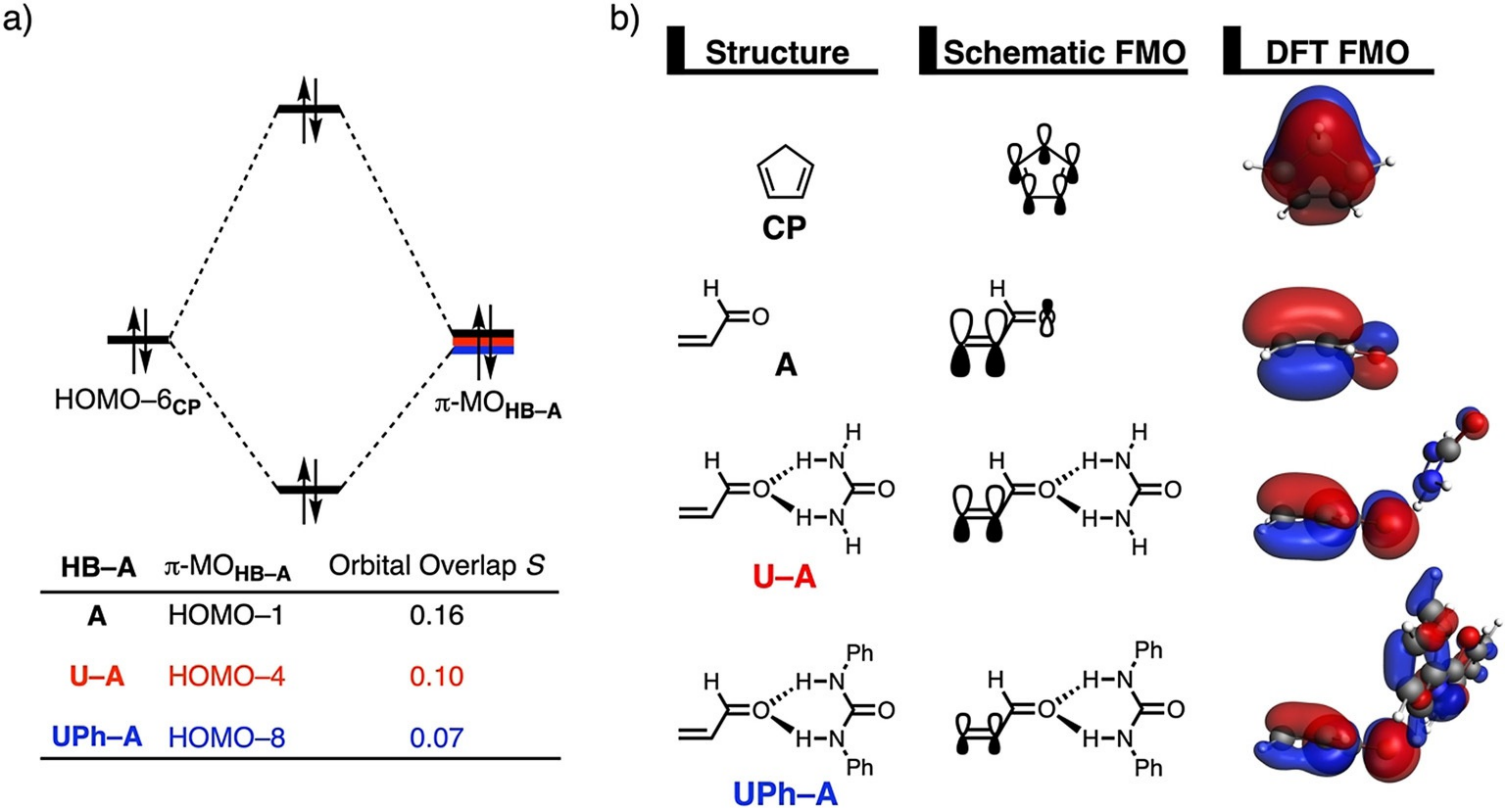
a) Molecular orbital diagram and the most significant occupied orbital overlaps of the *exo* Diels–Alder reactions between **CP** and **A**, **U‐A**, and **UPh‐A** and b) key occupied orbitals (isovalue = 0.03 au), computed at consistent geometries with a C_**CP**_⋅⋅⋅C_β_ bond length between **CP** and **HB‐A** of 2.14 Å at the ZORA‐M06‐2X/TZ2P//M06‐2X/def2‐SVPP level.

We used once again a Kohn–Sham molecular orbital (KS‐MO) analysis to rationalize why the Pauli repulsion becomes steadily less destabilizing when going from an uncatalyzed to a hydrogen bond donor‐catalyzed Diels–Alder reaction (Figure 7). The occupied molecular orbitals of **CP** and **A**, **U‐A**, and **UPh‐A** were quantified at consistent geometries with a C_**CP**_⋅⋅⋅C_β_ bond length between **CP** and **HB‐A** of 2.14 Å (Figure 7 a).^[31]^ The most important occupied π‐MO of the dienophile that are decisive for the trend in Pauli repulsion are the HOMO−1, HOMO−4, and HOMO−8 of **A**, **U‐A**, and **UPh‐A**, respectively, which are, in all cases, predominantly located on the reactive C=C double bond of **A**. The occupied orbital of **CP** involved in this interaction is the HOMO−6, where all 2p_π_ AOs and the σ_C‐H_ (pseudo‐π), located on the methylene bridge, are in‐phase. The orbital overlap between the occupied orbitals decreases from *S =* 0.16 for the uncatalyzed DA reaction to *S =* 0.10 and *S =* 0.07 for the reactions involving **U‐A** and **UPh‐A**, respectively (Figure 7 a). Coordination of a HB to **A** significantly polarizes the π‐orbital located on the C=C double bond of **A** towards the HB and away from the incoming **CP**, leading to a decreased occupied–occupied orbital overlap. The previously discussed donor–acceptor interaction between the two hydrogen bond donors of the HB and the hydrogen bond acceptor of **A** causes a charge transfer from **A** to the HB which results in less π‐orbital amplitude on the C=C double bond of **A** that points in the direction of the approaching **CP**. The replacement of the hydrogen atom of the parent urea **U** by a phenyl group in **UPh** notably increases the extent of charge transfer (from Δ*E*_oi_ = −3.2 kcal mol^−1^ for **U‐A** to Δ*E*_oi_ = −4.7 kcal mol^−1^ for **UPh‐A**; see Table 1) and, hence manifests in a progressively smaller π‐orbital amplitude on the C=C double bond and more orbital density on the HBs. This can clearly be seen when comparing the spatial distribution of the involved occupied orbitals in Figure 7 b. In addition, the larger degree of asynchronicity of the HB‐catalyzed DA reactions, which arises from a larger asymmetry in the π‐MO_**HB‐A**_ located on the reactive C=C double bond of the dienophile (vide supra), also plays a role in reducing the occupied–occupied orbital overlap between the reactants. As discussed above, a more asynchronous reaction has less orbital overlap at the α‐carbon of **HB‐A**, which, in turn, leads to less Pauli repulsion between the reactants and a lowering of the reaction barrier.

**Figure 8:**
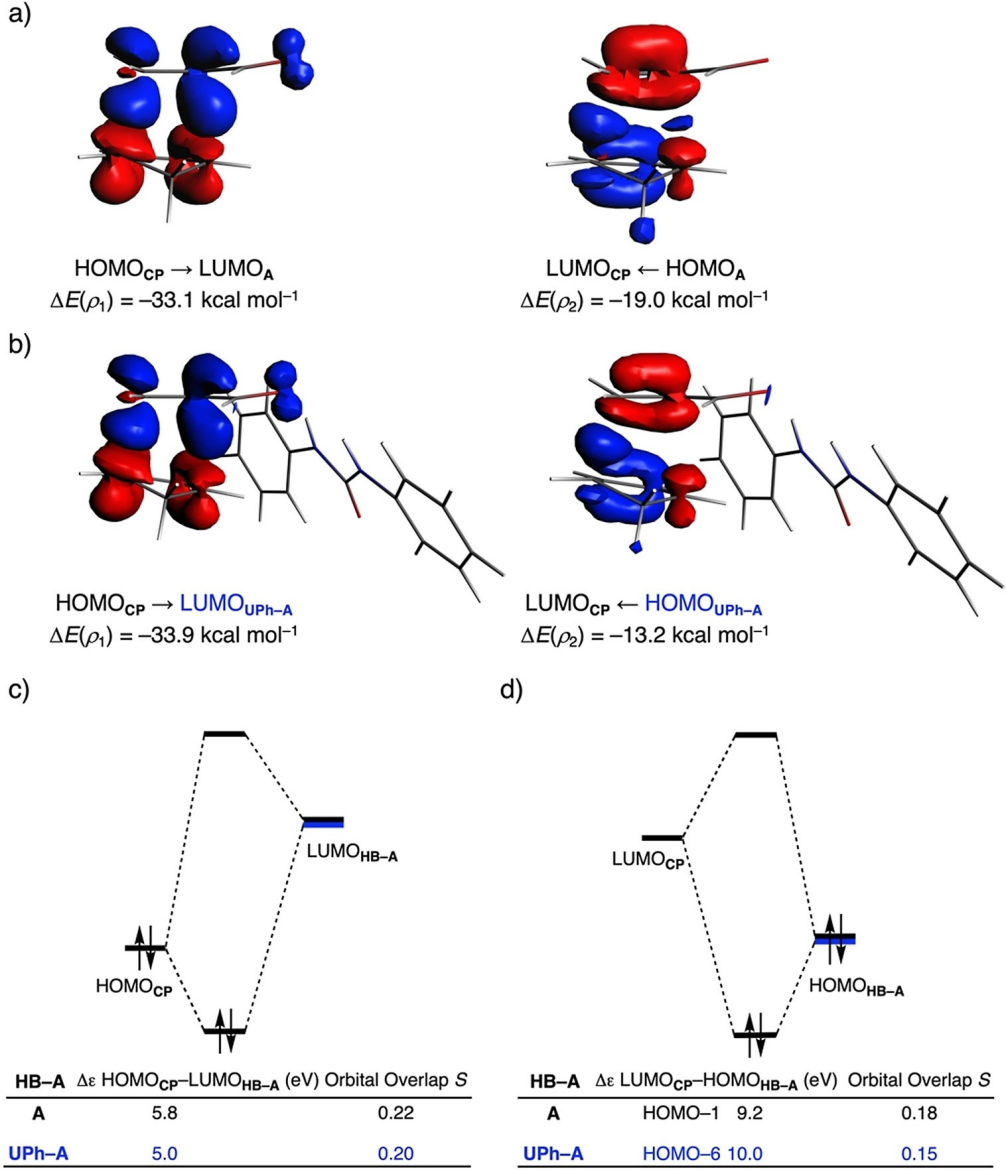
NOCV deformation densities Δ*ρ* (isovalue = 0.0015 au) and associated energies Δ*E*(*ρ*) for the normal electron demand (NED), HOMO_**CP**_ → LUMO_**HB‐A**_, and inverse electron demand (IED), LUMO_**CP**_ ← π‐HOMO_**HB‐A**_, where the color flow is red → blue, for the a) **A**, b) **UPh**; the Kohn–Sham molecular orbital analysis for the c) NED, and d) IED. All data computed, at consistent geometries with a C_**CP**_⋅⋅⋅C_β_ bond length between **CP** and **HB‐A** of 2.14 Å, at the ZORA‐M06‐2X/TZ2P//M06‐2X/def2‐SVPP level.

Finally, we address why the orbital interactions for the uncatalyzed DA reaction are more stabilizing than for the HB‐catalyzed counterpart despite the latter benefits from a smaller HOMO_**CP**_–LUMO_**HB‐A**_ gap (see Table 2). To this end, we applied the NOCV (natural orbitals for chemical valence)^[32]^ extension of the EDA method for the extreme situations represented by the uncatalyzed and **UPh**‐catalyzed Diels–Alder reactions. This approach identifies two main molecular orbital interactions that dominate the total orbital interactions, namely, the normal electron demand (NED) HOMO_**CP**_ → LUMO_**HB‐A**_ and the inverse electron demand (IED) LUMO_**CP**_ ← HOMO_**HB‐A**_ interactions (*ρ*_1_ and *ρ*_2_, respectively; see Figure 8 a and b). As expected for a NED Diels–Alder reaction, the former interaction is much stronger than the latter in both instances (Δ*E*(*ρ*_1_) > Δ*E*(*ρ*_2_)). Interestingly, whereas the NED interaction is only slightly stronger for the **UPh**‐catalyzed reaction (ΔΔ*E*(*ρ*_1_) = 0.8 kcal mol^−1^), the corresponding IED interaction is significantly weaker compared to the uncatalyzed reaction (ΔΔ*E*(*ρ*_2_) = −5.8 kcal mol^−1^). As a result, the total orbital interactions are, for the hydrogen bond donor‐catalyzed Diels–Alder reaction, less stabilizing than the uncatalyzed analogue. The mechanism behind these EDA‐NOCV results can be established by performing a Kohn–Sham molecular orbital analysis.[^12b^, ^28^] In line with the original rationale behind hydrogen bond donor catalysis,^[6]^ the HB catalyst decreases the NED HOMO_**CP**_–LUMO_**HB‐A**_ orbital energy gap from 5.8 eV for the uncatalyzed to 5.0 eV for the **UPh**‐catalyzed reaction (Figure 8 c). This reduction in orbital energy gap is large enough to overcome the slight decrease of orbital overlap, as a result of a more asynchronous reaction mode, and, therefore, coordination of a HB leads to a stronger HOMO_**CP**_–LUMO_**HB‐A**_ NED interaction. The IED interaction, however, is also modulated by the coordination of a HB. More specifically, the HB stabilizes all molecular orbital of **HB‐A** and hence also the HOMO_**HB‐A**_, manifesting in an IED orbital energy gap that increases from 9.2 eV for the uncatalyzed to 10.0 eV for the **UPh**‐catalyzed reaction (Figure 8 d). This, together with a reduced orbital overlap, results in a weaker IED interaction for the HB‐catalyzed compared to the uncatalyzed DA reaction. The weakening of the IED interaction effectively overrules the more stabilizing NED interaction and, for that reason, the total orbital interaction of **UPh**‐catalyzed DA reaction are less stabilizing than for the uncatalyzed DA reaction.

## Conclusions

Our theoretical study reveals that urea‐ and thiourea‐derived bifunctional hydrogen bond donating (HB) organocatalysts accelerate the Diels–Alder (DA) reaction between cyclopentadiene (**CP**) and acrolein (**A**) by coordinating to the carbonyl group of the dienophile through a double hydrogen bond and effectively lowering the reaction barrier up to 7 kcal mol^−1^. This catalytic effect is stronger for thiourea‐based HBs compared to their urea‐based counterparts. In addition, the *endo* selective uncatalyzed cycloaddition reaction becomes *exo* selective upon coordination of the HBs.

Our activation strain and Kohn–Sham molecular orbital analyses identified that the *exo* selective preference for the HB‐catalyzed DA reaction originates from both a larger degree of asynchronicity, which is induced by a more asymmetric π‐MO on the reactive C=C double bond of the dienophile, as well as a stronger C−H⋅⋅⋅π interaction between **CP** and HB along the *exo* pathway. This larger asynchronicity in the new C⋅⋅⋅C bond formation between the reactants along the *exo* pathway leads to two stabilizing and thus barrier lowering phenomena: (i) reduced occupied–occupied orbital overlap and hence more stabilizing interaction between the **CP** and **HB‐A**; and (ii) less destabilizing activation strain, as one newly forming C⋅⋅⋅C bond forms later than the other resulting in less pressure to deform the individual reactants.

The rate enhancement provoked by the HBs is exclusively caused by a diminished two‐center four‐electron Pauli repulsion between the occupied π‐orbitals of **CP** and **HB‐A** reactants. The reason for the reduced Pauli repulsion is the donor–acceptor interaction between the HB and **A**, which significantly polarizes the π‐orbital away from the reactive C=C double bond of **A**, resulting in less occupied–occupied orbital overlap with the incoming **CP**. Strikingly and in sharp contrast to the widely‐accepted rationale, we observed that, in the HB‐catalyzed DA reaction, the orbital interactions become less stabilizing compared to the uncatalyzed reaction. This is mainly due to a remarkable weakening of the inverse electron demand interaction, LUMO_**CP**_ ← π‐HOMO_**HB‐A**_, induced by the binding of the organocatalyst to the dienophile. Therefore, the results shown here demonstrate that the concept of *Pauli‐repulsion lowering catalysis* is a general phenomenon which is not only limited to conventional Lewis acid and iminium catalysis.

## Conflict of interest

The authors declare no conflict of interest.

## Supporting information

As a service to our authors and readers, this journal provides supporting information supplied by the authors. Such materials are peer reviewed and may be re‐organized for online delivery, but are not copy‐edited or typeset. Technical support issues arising from supporting information (other than missing files) should be addressed to the authors.

Supplementary

## References

[1a] F. Fringuelli , A. Taticchi in The Diels–Alder Reaction: Selected Practical Methods, Wiley, Hoboken, 2002. See also:

[1b] S. Sankararaman in Pericyclic Reactions-A Textbook: Reactions, Applications and Theory, Wiley-VCH, Weinheim, 2005.

[2] For reviews on the application of DA reactions in total synthesis, see:

[2a] K. C. Nicolaou , S. A. Snyder , T. Montagnon , G. Vassilikogiannakis , Angew. Chem. Int. Ed. 2002, 41, 1668–1698; 10.1002/1521-3773(20020517)41:10<1668::aid-anie1668>3.0.co;2-z 19750686

[2b] K.-I. Takao , R. Munakata , K.-i. Tadano , Chem. Rev. 2005, 105, 4779–4807; 16351062 10.1021/cr040632u

[2c] M. Juhl , D. Tanner , Chem. Soc. Rev. 2009, 38, 2983–2992. 19847335 10.1039/b816703f

[3] Representative examples:

[3a] D. Seebach , A. K. Beck , A. Heckel , Angew. Chem. Int. Ed. 2001, 40, 92–138; 11169693

[3b] P. M. Pihko , Angew. Chem. Int. Ed. 2004, 43, 2062–2064; 10.1002/anie.200301732 15083451

[3c] P. R. Schreiner , A. Wittkopp , Org. Lett. 2002, 4, 217–220; 11796054 10.1021/ol017117s

[3d] H. Jiang , C. Rodríguez-Escrich , T. K. Johansen , R. L. Davis , K. A. Jørgensen , Angew. Chem. Int. Ed. 2012, 51, 10271–10274; 10.1002/anie.201205836 22976506

[3e] A. Dieckmann , M. Breugst , K. N. Houk , J. Am. Chem. Soc. 2013, 135, 3237–3242; 23350816 10.1021/ja312043g

[3f] H. Jiang , D. C. Cruz , Y. Li , V. H. Lauridsen , K. A. Jørgensen , J. Am. Chem. Soc. 2013, 135, 5200–5207. 23473536 10.1021/ja4007244

[4] For recent reviews, see:

[4a] M. C. Gimeno , R. P. Herrera , Eur. J. Org. Chem. 2020, 1057–1068;

[4b] A. Skrzyńska , S. Frankowski , Ł. Albrecht , Asian J. Org. Chem. 2020, 9, 1688–1700, and references therein.

[5a] Y. Huang , V. H. Rawal , J. Am. Chem. Soc. 2002, 124, 9662–9663; 12175197 10.1021/ja0267627

[5b] Y. Huang , A. K. Unni , A. N. Thadani , V. H. Rawal , Nature 2003, 424, 146. 12853945 10.1038/424146a

[6a] A. Wittkopp , P. R. Schreiner , Chem. Eur. J. 2003, 9, 407–414; 12532289 10.1002/chem.200390042

[6b] P. R. Schreiner , Chem. Soc. Rev. 2003, 32, 289–296. See also, 14518182 10.1039/b107298f

[6c] Z. Zhang , P. R. Schreiner , Chem. Soc. Rev. 2009, 38, 1187–1198. 19421588 10.1039/b801793j

[7] A. Madarász , Z. Dósa , S. Varga , T. Soós , A. Csámpai , I. Pápai , ACS Catal. 2016, 6, 4379–4387.

[8a] P. Vermeeren , T. A. Hamlin , I. Fernández , F. M. Bickelhaupt , Angew. Chem. Int. Ed. 2020, 59, 6201–6206; 10.1002/anie.201914582 PMC7187354 31944503

[8b] P. Vermeeren , F. Brinkhuis , T. A. Hamlin , F. M. Bickelhaupt , Chem. Asian J. 2020, 15, 1167–1174. 32012430 10.1002/asia.202000009 PMC7187256

[9] P. Vermeeren , T. A. Hamlin , I. Fernández , F. M. Bickelhaupt , Chem. Sci. 2020, 11, 8105–8112. 10.1039/d0sc02901g PMC8163289 34094173

[10] T. A. Hamlin , I. Fernández , F. M. Bickelhaupt , Angew. Chem. Int. Ed. 2019, 58, 8922–8926; 10.1002/anie.201903196 PMC6617756 31033118

[11] For reviews, see:

[11a] I. Fernández , F. M. Bickelhaupt , Chem. Soc. Rev. 2014, 43, 4953–4967; 24699791 10.1039/c4cs00055b

[11b] F. M. Bickelhaupt , K. N. Houk , Angew. Chem. Int. Ed. 2017, 56, 10070–10086; 10.1002/anie.201701486 PMC5601271 28447369

[11c] I. Fernández , Chem. Sci. 2020, 11, 3769–3779. 10.1039/d0sc00222d PMC8152634 34122846

[11d] For a step-by-step protocol, see also: P. Vermeeren , S. C. C. van der Lubbe , C. Fonseca Guerra , F. M. Bickelhaupt , T. A. Hamlin , Nat. Protoc. 2020, 15, 649–667. 31925400 10.1038/s41596-019-0265-0

[12a] F. M. Bickelhaupt , E. J. Baerends , Reviews in Computational Chemistry, Vol. 15 (Eds.: K. B. Lipkowitz , D. B. Boyd ), Wiley-VCH, Weinheim, 2000, pp. 1–86;

[12b] R. van Meer , O. V. Gritsenko , E. J. Baerends , J. Chem. Theory Comput. 2014, 10, 4432–4441; 26588140 10.1021/ct500727c

[12c] L. Zhao , M. von Hopffgarten , D. M. Andrada , G. Frenking , WIREs Comput. Mol. Sci. 2018, 8, e1345 and references therein.

[13] Y. Zhao , D. G. Truhlar , Theor. Chem. Acc. 2008, 120, 215–241.

[14a] F. Weigend , R. Ahlrichs , Phys. Chem. Chem. Phys. 2005, 7, 3297–3305; 16240044 10.1039/b508541a

[14b] F. Weigend , Phys. Chem. Chem. Phys. 2006, 8, 1057. 16633586 10.1039/b515623h

[15] Gaussian 16, Revision B.01, M. J. Frisch, G. W. Trucks, H. B. Schlegel, G. E. Scuseria, M. A. Robb, J. R. Cheeseman, G. Scalmani, V. Barone, G. A. Petersson, H. Nakatsuji, X. Li, M. Caricato, A. V. Marenich, J. Bloino, B. G. Janesko, R. Gomperts, B. Mennucci, H. P. Hratchian, J. V. Ortiz, A. F. Izmaylov, J. L. Sonnenberg, D. Williams-Young, F. Ding, F. Lipparini, F. Egidi, J. Goings, B. Peng, A. Petrone, T. Henderson, D. Ranasinghe, V. G. Zakrzewski, J. Gao, N. Rega, G. Zheng, W. Liang, M. Hada, M. Ehara, K. Toyota, R. Fukuda, J. Hasegawa, M. Ishida, T. Nakajima, Y. Honda, O. Kitao, H. Nakai, T. Vreven, K. Throssell, J. A. Montgomery, Jr., J. E. Peralta, F. Ogliaro, M. J. Bearpark, J. J. Heyd, E. N. Brothers, K. N. Kudin, V. N. Staroverov, T. A. Keith, R. Kobayashi, J. Normand, K. Raghavachari, A. P. Rendell, J. C. Burant, S. S. Iyengar, J. Tomasi, M. Cossi, J. M. Millam, M. Klene, C. Adamo, R. Cammi, J. W. Ochterski, R. L. Martin, K. Morokuma, O. Farkas, J. B. Foresman, D. J. Fox, Gaussian, Inc., Wallingford CT, 2016.

[16] K. Fukui , Acc. Chem. Res. 1981, 14, 363–368.

[17] X. Sun , T. M. Soini , J. Poater , T. A. Hamlin , F. M. Bickelhaupt , J. Comput. Chem. 2019, 40, 2227–2233. 31165500 10.1002/jcc.25871 PMC6771738

[18a] G. te Velde , F. M. Bickelhaupt , E. J. Baerends , C. Fonseca Guerra , S. J. A. van Gisbergen , J. G. Snijders , T. Ziegler , J. Comput. Chem. 2001, 22, 931;

[18b] C. Fonseca Guerra, J. G. Snijders, G. te Velde, E. J. Baerends, *Theor. Chem. Acc*. **1998**, *99*, 391–403; (c) ADF2018.104, SCM Theoretical Chemistry, Vrije Universiteit: Amsterdam (Netherlands). http://www.scm.com.

[19a] E. van Lenthe , E. J. Baerends , J. Comput. Chem. 2003, 24, 1142–1156; 12759913 10.1002/jcc.10255

[19b] M. Franchini , P. H. T. Philipsen , E. van Lenthe , L. Visscher , J. Chem. Theory Comput. 2014, 10, 1994–2004. 26580526 10.1021/ct500172n

[20a] E. van Lenthe , E. J. Baerends , J. G. Snijders , J. Chem. Phys. 1993, 99, 4597–4610;

[20b] E. van Lenthe , E. J. Baerends , J. G. Snijders , J. Chem. Phys. 1994, 101, 9783–9792.

[21] E. G. Hohenstein , S. T. Chill , C. D. Sherrill , J. Chem. Theory Comput. 2008, 4, 1996–2000. 26620472 10.1021/ct800308k

[22] Y. Zhao , D. G. Truhlar , Acc. Chem. Res. 2008, 41, 157–167. 18186612 10.1021/ar700111a

[23a] F. Neese , WIREs Comput. Mol. Sci. 2018, 8, e1327;

[23b] C. Riplinger , B. Sandhoefer , A. Hansen , F. Neese , J. Chem. Phys. 2013, 139, 134101–134113. 24116546 10.1063/1.4821834

[24] E. R. Johnson , S. Keinan , P. Mori-Sánchez , J. Contreras-García , A. J. Cohen , W. Y. Yang , J. Am. Chem. Soc. 2010, 132, 6498–6506. 20394428 10.1021/ja100936w PMC2864795

[25] K. M. Lippert , K. Hof , D. Gerbig , D. Ley , H. Hausmann , S. Guenther , P. R. Schreiner , Eur. J. Org. Chem. 2012, 5919–5927.

[26] G. Jakab , C. Tancon , Z. Zhang , K. M. Lippert , P. R. Schreiner , Org. Lett. 2012, 14, 1724–1727. 22435999 10.1021/ol300307c

[27a] F. G. Bordwell , Acc. Chem. Res. 1988, 21, 456–463;

[27b] D. E. Gómez , L. Fabbrizzi , M. Licchelli , E. Monzani , Org. Biomol. Chem. 2005, 3, 1495–1500. 15827647 10.1039/b500123d

[28] This projection has been successfully used in related asynchronous Diels–Alder reactions (see refs. [8, 9, 11d]). See also the focus review: I. Fernández , F. M. Bickelhaupt , Chem. Asian J. 2016, 11, 3297–3304. 27863108 10.1002/asia.201601203

[29] T. A. Albright , J. K. Burdett , M.-H. Whangbo , Orbital Interactions in Chemistry, Wiley, 2013.

[30] Our results are in line with the experimental findings by Kramer and Bräse who reported a related highly *exo*-selective Diels–Alder reaction mediated by Schreiner's thiourea. See: C. S. Kramer , S. Bräse , Beilstein J. Org. Chem. 2013, 9, 1414–1418. 23946836 10.3762/bjoc.9.158 PMC3740680

[31] The consistent geometry structures with identical C_**CP**_⋅⋅⋅C_β_ bond lengths between **CP** and **HB-A** were obtained from IRC calculations.

[32] M. P. Mitoraj , A. Michalak , T. A. Ziegler , J. Chem. Theory Comput. 2009, 5, 962–975. 26609605 10.1021/ct800503d

