# Supplementary material for: Bifunctional Hydrogen Bond Donor‐Catalyzed Diels–Alder Reactions: Origin of Stereoselectivity and Rate Enhancement
Source: Chemistry. 2021 Jan 12;27(16):5180–90. doi: 10.1002/chem.202004496 (PMC8049058; doi:10.1002/chem.202004496)
Supplement: Supplementary file 1 — Supplementary [file CHEM-27-5180-s001.pdf]

Chemistry–A European Journal

Supporting Information

Bifunctional Hydrogen Bond Donor-Catalyzed Diels–Alder Reactions: Origin of Stereoselectivity and Rate Enhancement

Pascal Vermeeren,[a] Trevor A. Hamlin,*[a] F. Matthias Bickelhaupt,*[a, b] and Israel Fernández*[c]

Contents

Figure S1. Transition state structures for the uncatalyzed and hydrogen bond donor-catalyzed Diels–Alder reaction between **CP** and **A** forming an *endo* (left) or *exo* (right) cycloadduct, computed at M06-2X/def2-def2-SVPP.

Figure S2. Activation barriers ($\Delta E^{\ddagger}$) for the uncatalyzed and HB-catalyzed Diels-Alder reactions versus the HOMO$_{\mathbf{CP}}$–LUMO$_{\mathbf{HB–A}}$ energy gap ($\Delta\varepsilon_{\text{H–L}}$) of the reactants for the a) *endo* and b) *exo* pathway, computed at ZORA-M06-2X/TZ2P//M06-2X/def2-SVPP.

Table S1. Energy decomposition analyses (in kcal mol^{-1}) of the interaction between **CP** and the HB at the transition states of the *endo* and *exo* pathway of the interaction between **CP** and **HB–A**.

Figure S3. a) Activation strain analyses and b) energy decomposition analyses of the catalyzed *endo* and *exo* Diels-Alder reactions between **CP** and **A**, where the energy values are projected onto the shorter newly forming $C_{\mathbf{CP}}$•••C_{β} bond between **CP** and **A**, computed at ZORA-M06-2X/TZ2P//M06-2X/def2-SVPP.

Figure S4. a) Activation strain analyses and b) energy decomposition analyses of the catalyzed *endo* and *exo* Diels-Alder reactions between **CP** and **U–A**, where the energy values are projected onto the shorter newly forming $C_{\mathbf{CP}}$•••C_{β} bond between **CP** and **U–A**, computed at ZORA-M06-2X/TZ2P//M06-2X/def2-SVPP.

Figure S5. a) Activation strain analyses and b) energy decomposition analyses of the catalyzed *endo* and *exo* Diels-Alder reactions between **CP** and **TU–A**, where the energy values are projected onto the shorter newly forming $C_{\mathbf{CP}}$•••C_{β} bond between **CP** and **TU–A**, computed at ZORA-M06-2X/TZ2P//M06-2X/def2-SVPP.

Figure S6. a) Activation strain analyses and b) energy decomposition analyses of the catalyzed *endo* and *exo* Diels-Alder reactions between **CP** and **TUPh-A**, where the energy values are projected onto the shorter newly forming $C_{\mathbf{CP}}$•••C_{β} bond between **CP** and **TUPh–A**, computed at ZORA-M06-2X/TZ2P//M06-2X/def2-SVPP.

Figure S7. Occupied-occupied orbital overlap between HOMO–6$_{\mathbf{CP}}$ and HOMO–6$_{\mathbf{UPh\text{-}A}}$ for the *endo* and *exo* Diels-Alder reactions between **CP** and **UPh–A** going from the reactants to the transition state, where the orbital overlap value is projected onto the shorter newly forming $C_{\mathbf{CP}}$•••C_{β} bond between **CP** and the **UPh–A**, computed at ZORA-M06-2X/TZ2P//M06-2X/def2-SVPP.

Table S2. Occupied-occupied orbital overlap for the *endo* Diels-Alder reactions between **CP** and **UPh–A** in the transition state.

Table S3. Occupied-occupied orbital overlap for the *exo* Diels-Alder reactions between **CP** and **UPh–A** in the transition state.

Figure S8. a) Activation strain analyses and b) energy decomposition analyses of the *endo* Diels-Alder reactions between **CP** and **U–A** and **TU–A**, where the energy values are projected onto the shorter newly forming $C_{\mathbf{CP}}$•••C_{β} bond between **CP** and **HB–A**, computed at ZORA-

M06-2X/TZ2P//M06-2X/def2-SVPP.

Figure S9. a) Activation strain analyses and b) energy decomposition analyses of the *endo* Diels-Alder reactions between **CP** and **UPh–A** and **TUPh–A**, where the energy values are projected onto the shorter newly forming C_{CP}•••C_{β} bond between **CP** and **HB–A**, computed at ZORA-M06-2X/TZ2P//M06-2X/def2-SVPP.

Figure S10. a) Activation strain analyses and b) energy decomposition analyses of the *endo* Diels-Alder reactions between **CP** and **UPh–A** and **TUPh–A**, where the energy values are projected onto the shorter newly forming C_{CP}•••C_{β} bond between **CP** and **HB–A**, computed at ZORA-M06-2X/TZ2P//M06-2X/def2-SVPP.

Figure S11. a) Activation strain analyses and b) energy decomposition analyses of the *endo* Diels-Alder reactions between **CP** and **A**, **U–A**, and **UPh–A**, where the energy values are projected onto the shorter newly forming C_{CP}•••C_{β} bond between **CP** and **HB–A**, computed at ZORA-M06-2X/TZ2P//M06-2X/def2-SVPP.

Figure S12. a) Activation strain analyses and b) energy decomposition analyses of the *endo* Diels-Alder reactions between **CP** and **A**, **TU–A**, and **TUPh–A**, where the energy values are projected onto the shorter newly forming C_{CP}•••C_{β} bond between **CP** and **HB–A**, computed at ZORA-M06-2X/TZ2P//M06-2X/def2-SVPP.

Figure S13. a) Activation strain analyses and b) energy decomposition analyses of the *exo* Diels-Alder reactions between **CP** and **A**, **TU–A**, and **TUPh–A**, where the energy values are projected onto the shorter newly forming C_{CP}•••C_{β} bond between **CP** and **HB–A**, computed at ZORA-M06-2X/TZ2P//M06-2X/def2-SVPP.

Figure S14. Key occupied orbitals (isovalue = 0.03 au) computed at the equilibrium structures of **A**, **U–A**, and **UPh–A**, where the MO coefficients of the carbon $2p_z$ atomic orbitals, contributing to the occupied orbitals, are shown. Computed at the ZORA-M06-2X/TZ2P//M06-2X/def2-SVPP level.

Table S2. Cartesian coordinates (in Å), energies (in kcal mol^{-1}), and number of imaginary frequencies of all stationary points, computed at M06-2X/def2-SVPP.

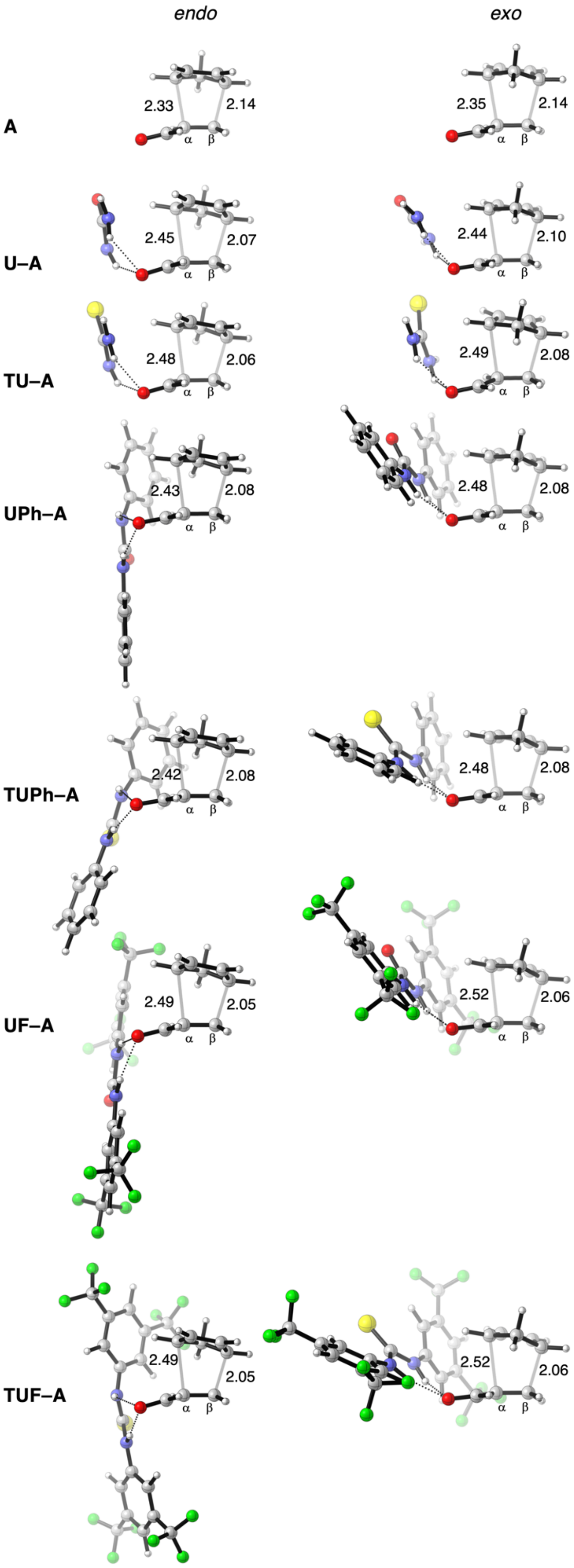

Figure S1. Transition state structures for the uncatalyzed and hydrogen bond donor-catalyzed Diels–Alder reaction between **CP** and **A** forming an *endo* (left) or *exo* (right) cycloadduct, computed at M06-2X/def2-def2-SVPP.

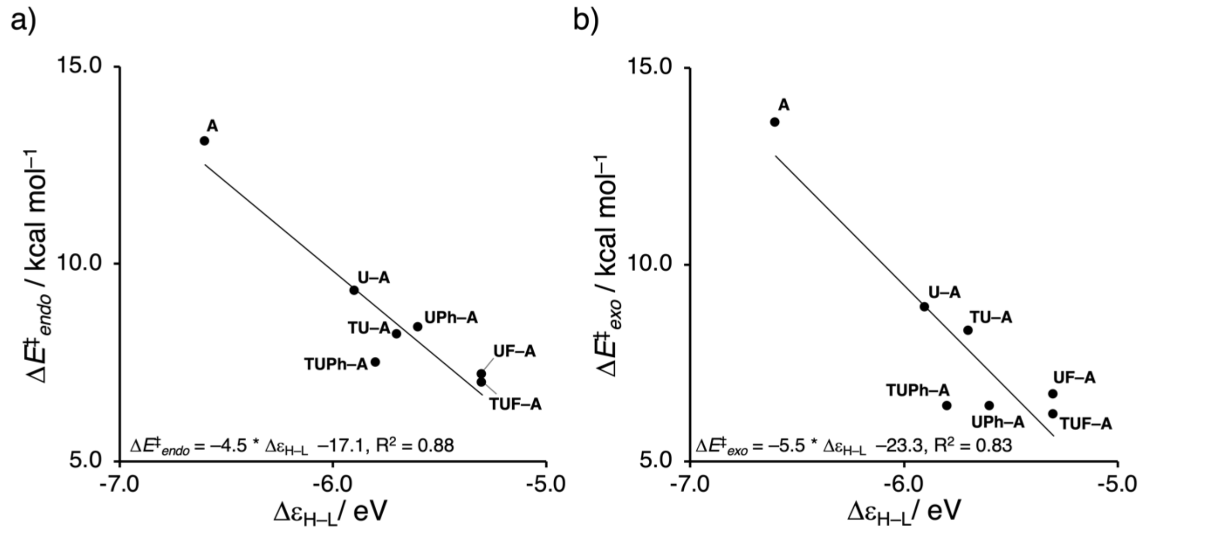

Figure S2. Activation barriers ($\Delta E^{\ddagger}$) for the uncatalyzed and HB-catalyzed Diels-Alder reactions versus the HOMO_{CP}–$\text{LUMO}_{\text{HB–A}}$ energy gap ($\Delta\varepsilon_{\text{H–L}}$) of the reactants for the a) *endo* and b) *exo* pathway, computed at ZORA-M06-2X/TZ2P//M06-2X/def2-SVPP.

Table S1. Energy decomposition analyses (in kcal mol^{-1}) of the interaction between **CP** and the HB at the transition states structures of the *endo* and *exo* pathway for the hydrogen bond donor-catalyzed Diels–Alder reaction.

HB	cycloadduct	ΔE_{int}	ΔV_{elstat}	ΔE_{Pauli}	ΔE_{oi}
**U**	*endo*	–1.4	–2.7	2.5	–1.2
	exo	–1.1	–2.9	3.0	–1.2
**TU**	*endo*	–2.2	–3.2	2.7	–1.7
	exo	–2.4	–3.9	3.2	–1.8
**UPh**	*endo*	–2.7	–2.7	1.3	–1.3
	exo	–3.3	–3.5	2.1	–1.8
**TUPh**	*endo*	–3.1	–3.0	1.6	–1.6
	exo	–3.5	–3.7	2.2	–1.9
**UF**	*endo*	–2.1	–2.3	1.3	–1.1
	exo	–3.6	–3.0	1.4	–2.0
**TUF**	*endo*	–3.7	–3.9	2.3	–2.0
	exo	–3.7	–3.0	1.4	–2.0

[a] Computed at ZORA-M06-2X/TZ2P//M06-2X/def2-SVPP

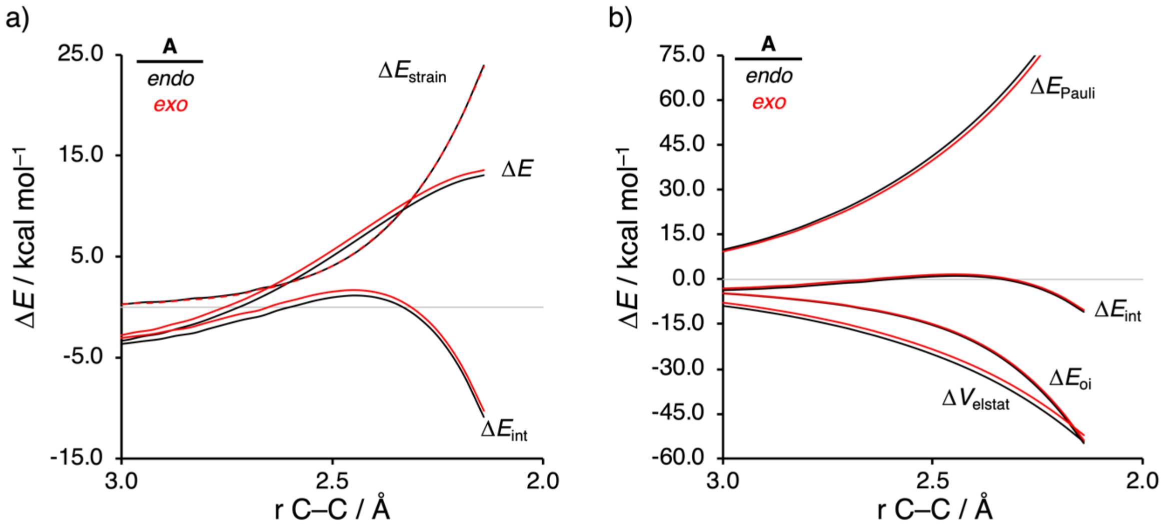

Figure S3. a) Activation strain analyses and b) energy decomposition analyses of the catalyzed *endo* and *exo* Diels-Alder reactions between **CP** and **A**, where the energy values are projected onto the shorter newly forming C_{CP}•••C_{β} bond between **CP** and **A**, computed at ZORA-M06-2X/TZ2P//M06-2X/def2-SVPP.

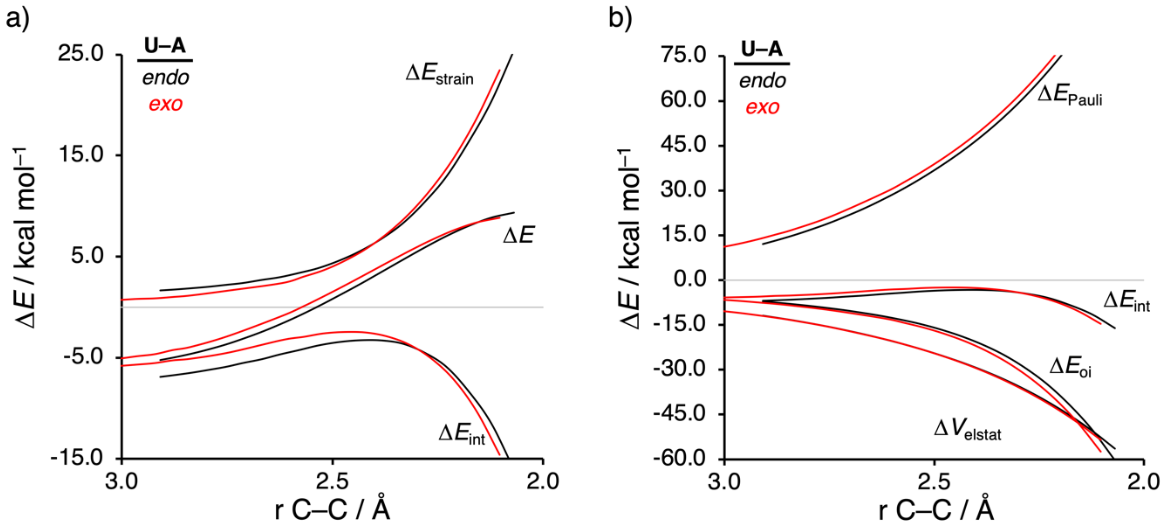

Figure S4. a) Activation strain analyses and b) energy decomposition analyses of the catalyzed *endo* and *exo* Diels-Alder reactions between **CP** and **U–A**, where the energy values are projected onto the shorter newly forming C_{CP}•••C_{β} bond between **CP** and **U–A**, computed at ZORA-M06-2X/TZ2P//M06-2X/def2-SVPP.

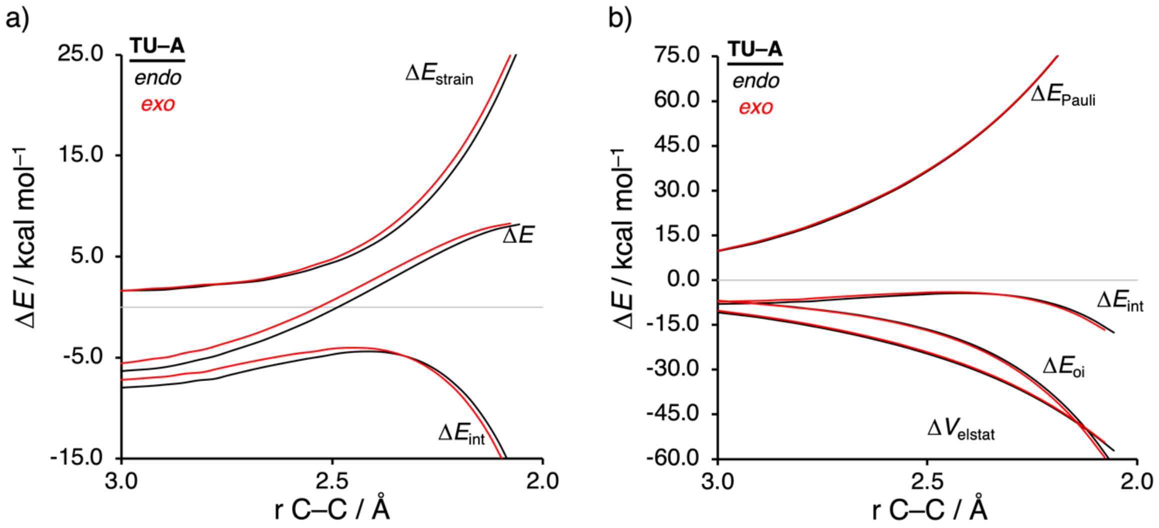

Figure S5. a) Activation strain analyses and b) energy decomposition analyses of the catalyzed *endo* and *exo* Diels-Alder reactions between **CP** and **TU–A**, where the energy values are projected onto the shorter newly forming C_{CP}•••C_{β} bond between **CP** and **TU–A**, computed at ZORA-M06-2X/TZ2P//M06-2X/def2-SVPP.

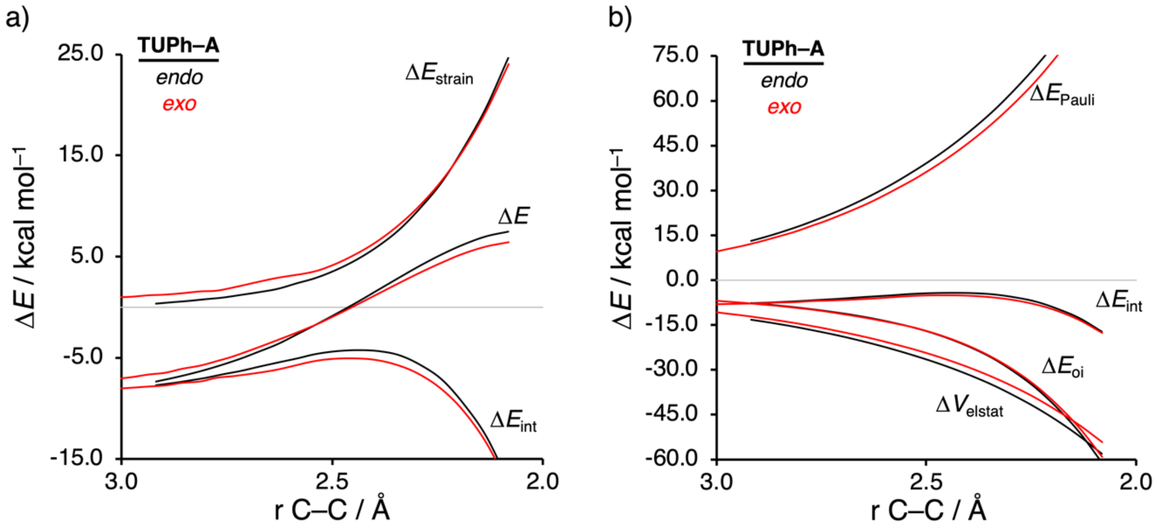

Figure S6. a) Activation strain analyses and b) energy decomposition analyses of the catalyzed *endo* and *exo* Diels-Alder reactions between **CP** and **TUPh-A**, where the energy values are projected onto the shorter newly forming C_{CP}•••C_{β} bond between **CP** and **TUPh–A**, computed at ZORA-M06-2X/TZ2P//M06-2X/def2-SVPP.

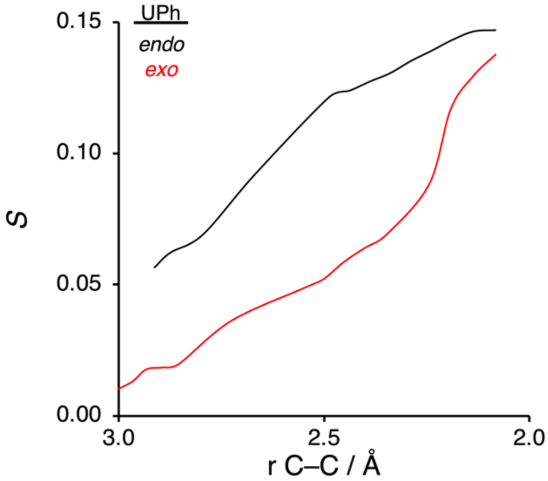

Figure S7. Occupied-occupied orbital overlap between HOMO–$6_{\mathbf{CP}}$ and HOMO–$6_{\mathbf{UPh\text{-}A}}$ for the *endo* and *exo* Diels-Alder reactions between **CP** and **UPh–A** going from the reactants to the transition state, where the orbital overlap value is projected onto the shorter newly forming $C_{\mathbf{CP}}$•••C_{β} bond between **CP** and the **UPh–A**, computed at ZORA-M06-2X/TZ2P//M06-2X/def2-SVPP.

Table S2. Occupied-occupied orbital overlap for the *endo* Diels-Alder reactions between **CP** and **UPh–A** in the transition state.[a]

							**UPh-A**					
		HOMO	HOMO–1	HOMO–2	HOMO–3	HOMO–4	HOMO–5	HOMO–6	HOMO–7	HOMO–8	HOMO–9	HOMO–10
**CP**	HOMO	0.01	0.01	0.00	0.00	0.01	0.00	0.05	0.01	0.00	0.00	0.00
	HOMO–1	0.01	0.01	0.00	0.01	0.01	0.03	0.10	0.03	0.01	0.01	0.00
	HOMO–2	0.00	0.00	0.00	0.00	0.00	0.00	0.01	0.00	0.00	0.00	0.01
	HOMO–3	0.00	0.00	0.00	0.01	0.00	0.02	0.00	0.00	0.02	0.00	0.01
	HOMO–4	0.01	0.01	0.00	0.02	0.00	0.00	0.02	0.01	0.01	0.00	0.00
	HOMO–5	0.01	0.01	0.00	0.03	0.00	0.01	0.01	0.01	0.00	0.00	0.01
	HOMO–6	0.01	0.01	0.00	0.01	0.01	0.04	0.15	0.04	0.00	0.01	0.00
	HOMO–7	0.01	0.01	0.00	0.02	0.00	0.00	0.03	0.01	0.01	0.00	0.00
	HOMO–8	0.01	0.01	0.00	0.03	0.00	0.00	0.00	0.00	0.01	0.00	0.00
	HOMO–9	0.00	0.00	0.00	0.02	0.01	0.03	0.05	0.00	0.01	0.00	0.00
	HOMO–10	0.01	0.01	0.00	0.02	0.00	0.01	0.04	0.01	0.01	0.00	0.00

[a] Computed at ZORA-M06-2X/TZ2P//M06-2X/def2-SVPP

Table S3. Occupied-occupied orbital overlap for the *exo* Diels-Alder reactions between **CP** and **UPh–A** in the transition state.[a]

							**UPh-A**					
		HOMO	HOMO–1	HOMO–2	HOMO–3	HOMO–4	HOMO–5	HOMO–6	HOMO–7	HOMO–8	HOMO–9	HOMO–10
**CP**	HOMO	0.00	0.00	0.00	0.03	0.00	0.00	0.05	0.00	0.01	0.00	0.00
	HOMO–1	0.00	0.00	0.00	0.02	0.00	0.02	0.10	0.00	0.03	0.01	0.00
	HOMO–2	0.00	0.00	0.00	0.00	0.00	0.02	0.01	0.02	0.01	0.00	0.00
	HOMO–3	0.00	0.00	0.00	0.00	0.00	0.01	0.02	0.01	0.02	0.00	0.01
	HOMO–4	0.01	0.01	0.00	0.00	0.00	0.01	0.02	0.02	0.00	0.00	0.00
	HOMO–5	0.00	0.00	0.00	0.01	0.00	0.00	0.01	0.00	0.01	0.00	0.00
	HOMO–6	0.00	0.01	0.00	0.00	0.00	0.02	0.14	0.01	0.00	0.01	0.00
	HOMO–7	0.01	0.00	0.00	0.01	0.00	0.01	0.02	0.00	0.01	0.00	0.00
	HOMO–8	0.01	0.01	0.00	0.00	0.00	0.01	0.01	0.02	0.00	0.00	0.00
	HOMO–9	0.00	0.00	0.00	0.01	0.00	0.02	0.05	0.02	0.01	0.00	0.00
	HOMO–10	0.01	0.01	0.00	0.00	0.00	0.00	0.04	0.01	0.01	0.00	0.00

[a] Computed at ZORA-M06-2X/TZ2P//M06-2X/def2-SVPP

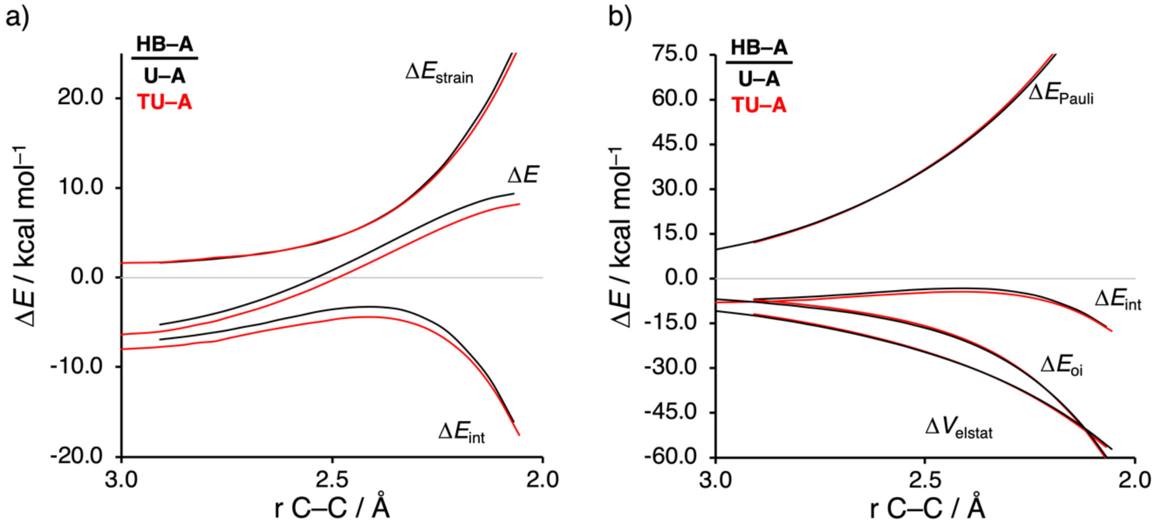

Figure S8. a) Activation strain analyses and b) energy decomposition analyses of the *endo* Diels-Alder reactions between **CP** and **U–A** and **TU–A**, where the energy values are projected onto the shorter newly forming C_{CP}•••C_{β} bond between **CP** and **HB–A**, computed at ZORA-M06-2X/TZ2P//M06-2X/def2-SVPP.

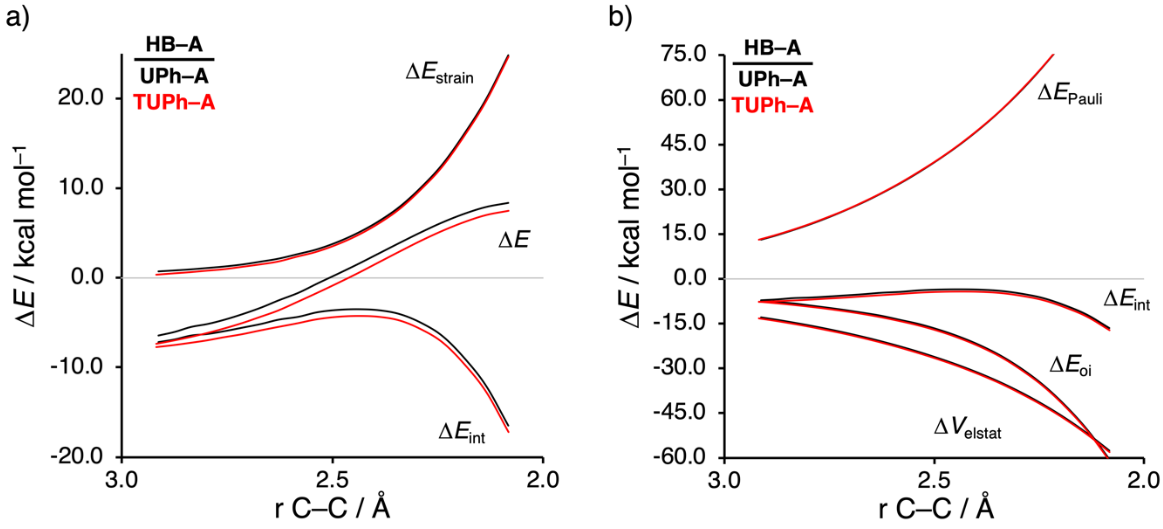

Figure S9. a) Activation strain analyses and b) energy decomposition analyses of the *endo* Diels-Alder reactions between **CP** and **UPh–A** and **TUPh–A**, where the energy values are projected onto the shorter newly forming C_{CP}•••C_{β} bond between **CP** and **HB–A**, computed at ZORA-M06-2X/TZ2P//M06-2X/def2-SVPP.

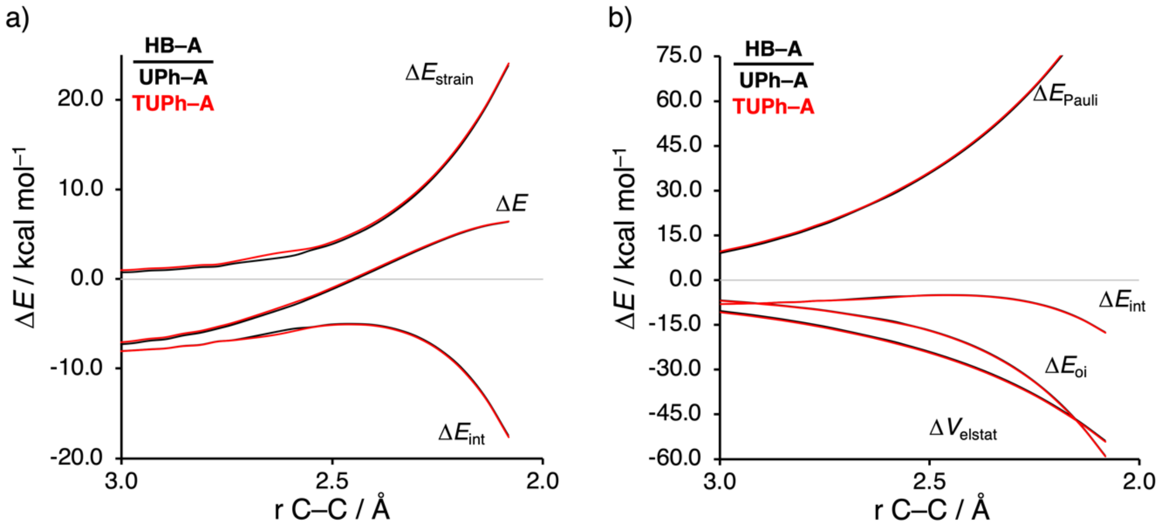

Figure S10. a) Activation strain analyses and b) energy decomposition analyses of the *endo* Diels-Alder reactions between **CP** and **UPh–A** and **TUPh–A**, where the energy values are projected onto the shorter newly forming C_{CP}•••C_{β} bond between **CP** and **HB–A**, computed at ZORA-M06-2X/TZ2P//M06-2X/def2-SVPP.

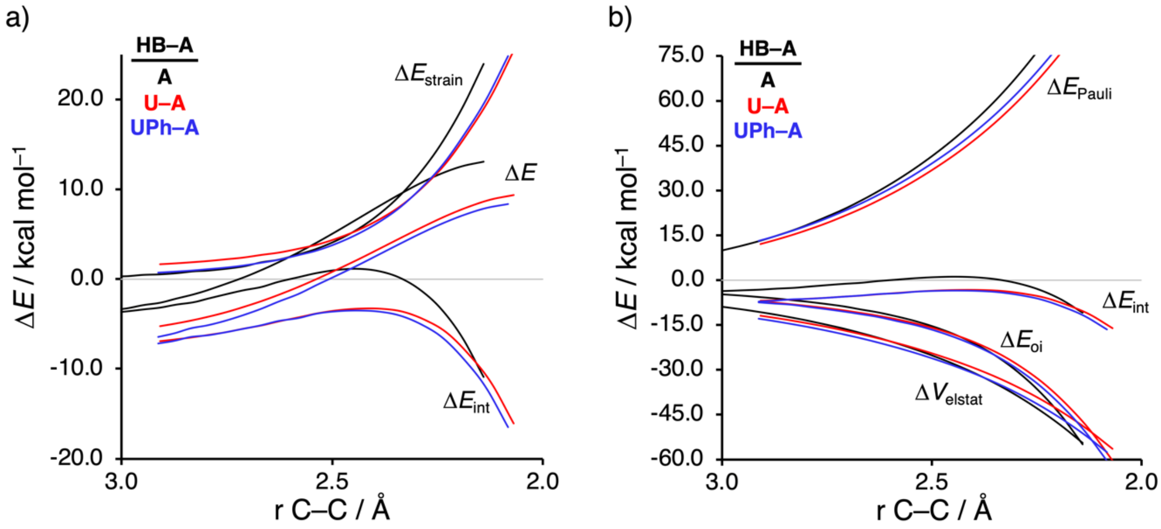

Figure S11. a) Activation strain analyses and b) energy decomposition analyses of the *endo* Diels-Alder reactions between **CP** and **A**, **U–A**, and **UPh–A**, where the energy values are projected onto the shorter newly forming C_{CP}•••C_{β} bond between **CP** and **HB–A**, computed at ZORA-M06-2X/TZ2P//M06-2X/def2-SVPP.

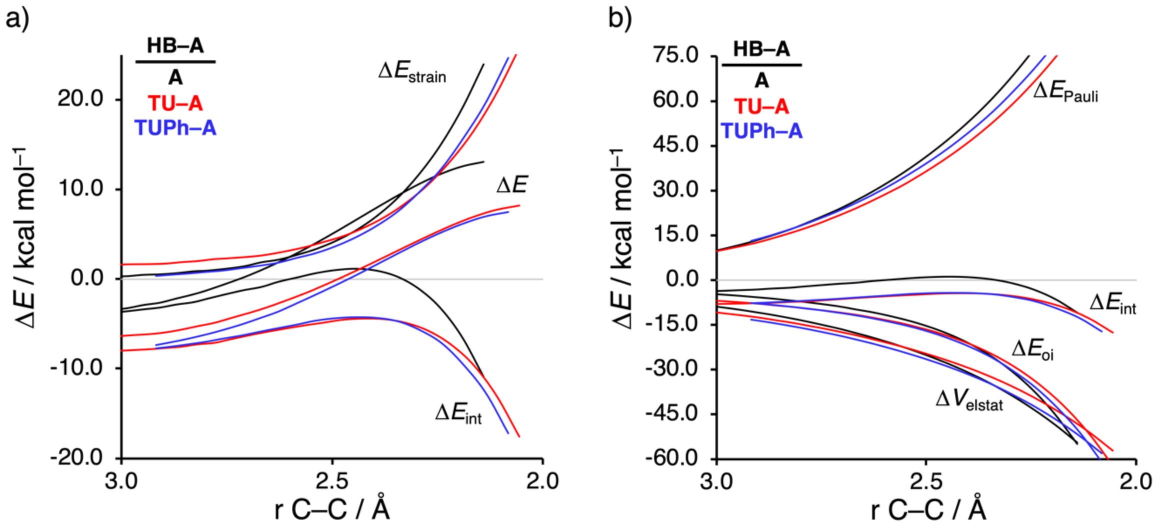

Figure S12. a) Activation strain analyses and b) energy decomposition analyses of the *endo* Diels-Alder reactions between **CP** and **A**, **TU–A**, and **TUPh–A**, where the energy values are projected onto the shorter newly forming C_{CP}•••C_{β} bond between **CP** and **HB–A**, computed at ZORA-M06-2X/TZ2P//M06-2X/def2-SVPP.

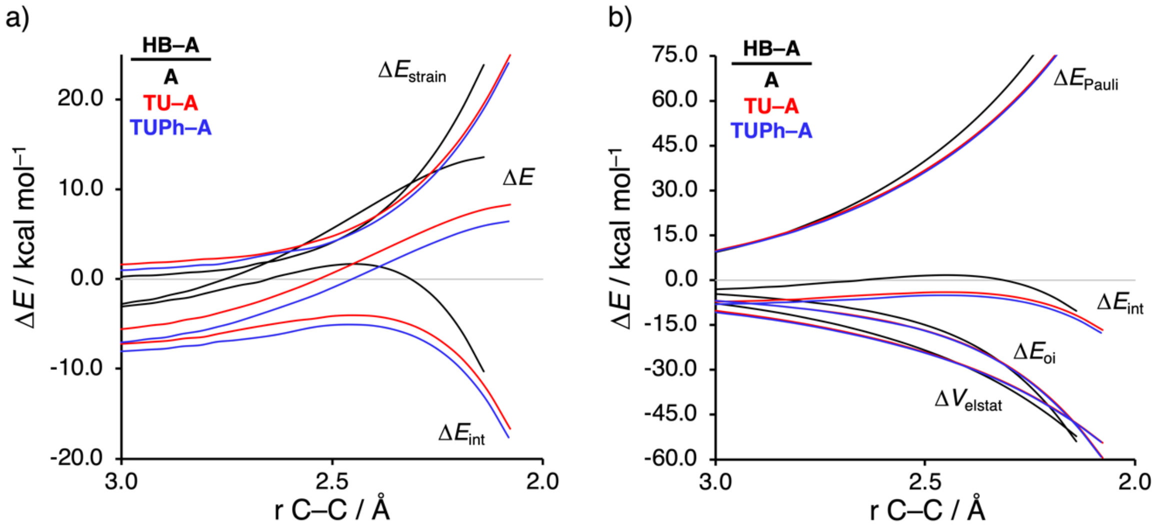

Figure S13. a) Activation strain analyses and b) energy decomposition analyses of the *exo* Diels-Alder reactions between **CP** and **A**, **TU–A**, and **TUPh–A**, where the energy values are projected onto the shorter newly forming C_{CP}•••C_{β} bond between **CP** and **HB–A**, computed at ZORA-M06-2X/TZ2P//M06-2X/def2-SVPP.

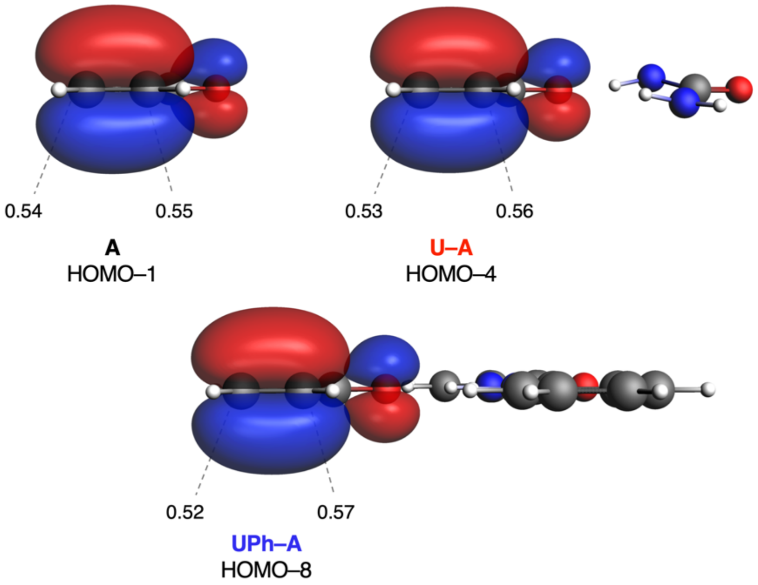

Figure S14. Key occupied orbitals (isovalue = 0.03 au) computed at the equilibrium structures of **A**, **U–A**, and **UPh–A**, where the MO coefficients of the carbon 2p_z atomic orbitals, contributing to the occupied orbitals, are shown. Computed at the ZORA-M06-2X/TZ2P//M06-2X/def2-SVPP level.

Table S4. Cartesian coordinates (in Å), energies (in Hartree), and number of imaginary frequencies of all stationary points, computed at M06-2X/def2-SVPP.

cyclopentadiene (CP)
E = −193.757376
H = −193.752288
G = −193.783979
N_{imag} = 0

C	1.17779	0.28251	0.00000
C	0.73558	-0.99032	-0.00000
C	-0.73557	-0.99032	-0.00000
C	-1.17779	0.28250	0.00000
C	-0.00000	1.21499	0.00000
H	2.21842	0.61047	0.00000
H	1.35660	-1.88803	-0.00000
H	-1.35659	-1.88804	-0.00000
H	-2.21842	0.61047	0.00000
H	-0.00000	1.87948	-0.88521
H	-0.00000	1.87948	0.88521

Acrolein (A)
E = −191.606101
H = −191.60080
G = −191.632382
N_{imag} = 0

O	1.78657	-0.12203	0.00002
C	0.68162	0.34839	-0.00017
H	0.52268	1.45788	0.00023
C	-0.56388	-0.45242	-0.00004
C	-1.75805	0.14461	0.00010
H	-0.44503	-1.54099	-0.00008
H	-2.69320	-0.42352	0.00018
H	-1.83515	1.23938	0.00014

Acrolein + Urea (U-A)
E = −416.538391
H = −416.526644
G = −416.577863
N_{imag} = 0

O	-0.89256	0.39083	-0.06114
C	-2.08490	0.57332	-0.09185
H	-2.49728	1.59630	-0.26567
C	-3.09062	-0.48904	0.08894
C	-4.39410	-0.19840	0.03941
H	-2.71636	-1.50313	0.26109
H	-5.16404	-0.96461	0.16926
H	-4.73346	0.83006	-0.13540
H	1.06611	1.26981	-0.09567
H	0.75135	-0.98067	0.04750
N	1.73896	-1.12761	-0.13099
C	2.63730	-0.09522	0.01045

H	2.12530	-2.05750	-0.03242
N	2.04906	1.14291	0.12085
H	2.67692	1.93271	0.04571
O	3.83871	-0.26258	0.03550

Acrolein + 1,3-diphenylurea (UPh-A)

E = −877.943360
H = −877.923144
G = −877.996.417
N_{imag} = 0

O	0.24256	2.41639	0.00045
C	0.38533	3.61675	-0.00017
H	-0.50506	4.28855	-0.00112
C	1.69161	4.29393	0.00027
C	1.75451	5.62937	-0.00037
H	2.58368	3.65954	0.00118
H	2.70914	6.16354	-0.00003
H	0.83994	6.23505	-0.00128
H	-1.02094	0.79968	-0.00042
H	0.98751	0.50314	0.00118
N	0.96579	-0.51305	0.00084
C	-0.27864	-1.11705	-0.00031
N	-1.29461	-0.17945	-0.00081
O	-0.45478	-2.31561	-0.00087
C	2.21888	-1.13545	0.00043
C	2.41080	-2.52627	0.00116
C	3.34549	-0.29519	-0.00054
C	3.70723	-3.03978	0.00087
C	4.63123	-0.82365	-0.00080
C	4.82372	-2.20526	-0.00011
H	1.54825	-3.18781	0.00195
H	3.19900	0.78972	-0.00115
H	3.84017	-4.12482	0.00145
H	5.48985	-0.14727	-0.00156
H	5.83185	-2.62506	-0.00029
C	-2.67463	-0.41225	-0.00039
C	-3.50962	0.71824	0.00064
C	-3.26032	-1.68818	-0.00105
C	-4.89328	0.58361	0.00101
C	-4.64978	-1.80510	-0.00061
C	-5.47711	-0.68329	0.00041
H	-3.05677	1.71479	0.00121
H	-2.62605	-2.57090	-0.00191
H	-5.51936	1.47956	0.00180
H	-5.09082	-2.80536	-0.00112
H	-6.56356	-0.79370	0.00071

Acrolein + 1,3-bis(3,5-bis(trifluoromethyl)phenyl)urea (UF-A)

E = −2224.693060

H = −2224.685437

G = −2224.769955

N_{imag} = 0

O	0.17154	3.12409	-0.01228
C	0.36437	4.31114	-0.15950
H	-0.49910	5.01517	-0.18834
C	1.69012	4.92676	-0.30968
C	1.79385	6.25196	-0.46003
H	2.56608	4.26978	-0.29573
H	2.76357	6.74500	-0.57494
H	0.90102	6.88908	-0.47243
H	-1.05293	1.44027	0.06882
H	0.96855	1.34129	0.07791
N	1.04841	0.32615	0.06554
C	-0.12763	-0.39942	0.04717
N	-1.23017	0.43785	0.05911
O	-0.18496	-1.60720	0.02475
C	2.34979	-0.16697	0.04985
C	2.68604	-1.52901	-0.03243
C	3.37892	0.78211	0.10968
C	4.02867	-1.89331	-0.06036
C	4.71163	0.39109	0.07960
C	5.05764	-0.95308	-0.01078
H	1.90333	-2.28298	-0.07980
H	3.12590	1.84293	0.17591
H	6.10394	-1.26306	-0.04886
C	-2.57272	0.06201	0.04405
C	-3.52309	1.09838	0.03469
C	-3.02020	-1.26541	0.03866
C	-4.87952	0.81243	0.02001
C	-4.39181	-1.52369	0.01905
C	-5.33676	-0.50609	0.00872
H	-3.19015	2.13921	0.03506
H	-2.30286	-2.08419	0.04837
H	-6.40419	-0.73075	-0.00967
C	5.78202	1.44410	0.19495
F	5.37840	2.61117	-0.32361
F	6.10321	1.67910	1.46837
F	6.89795	1.08554	-0.43507
C	4.41115	-3.35086	-0.13833
F	5.25525	-3.57092	-1.14915
F	5.03214	-3.74349	0.97629
F	3.35561	-4.14054	-0.31161
C	-4.82731	-2.96761	-0.01689
F	-6.14811	-3.09060	0.11730
F	-4.48607	-3.54638	-1.16908
F	-4.25353	-3.67111	0.95891
C	-5.88656	1.93211	0.05787
F	-5.33872	3.10520	-0.26954

F	-6.89572	1.70718	-0.78304
F	-6.41543	2.06991	1.27509

Acrolein + Thioruea (TU-A)

E = −739.441708
H = −739.429644
G = −739.481619
N_{imag} = 0

O	-1.42544	0.42971	-0.00186
C	-2.62416	0.57790	-0.00013
H	-3.06616	1.60261	0.00238
C	-3.59504	-0.52945	-0.00100
C	-4.90704	-0.27382	0.00131
H	-3.19049	-1.54650	-0.00354
H	-5.65278	-1.07420	0.00078
H	-5.27819	0.75845	0.00388
H	0.48485	1.33653	0.00085
H	0.19843	-0.90372	-0.00100
N	1.20474	-1.04489	0.00117
C	2.06907	-0.00479	0.00037
H	1.60153	-1.97472	-0.00035
N	1.49439	1.21904	0.00003
H	2.11170	2.01974	0.00001
S	3.72773	-0.21837	0.00001

Acrolein + 1,3-diphenylthiourea (TUPh-A)

E = −1200.835995
H = −1200.815480
G = −1200.889366
N_{imag} = 0

O	0.18236	2.57663	0.77899
C	0.87430	3.28903	0.08679
H	0.54819	4.33202	-0.13970
C	2.15905	2.88561	-0.50741
C	2.84921	3.75699	-1.25101
H	2.51383	1.86487	-0.32169
H	3.80538	3.49047	-1.71069
H	2.47219	4.77256	-1.42437
H	-1.10303	0.96237	0.65760
H	0.77561	0.58920	0.90162
N	0.81989	-0.30210	0.40914
C	-0.39529	-0.84427	0.06827
N	-1.40361	0.03130	0.37330
S	-0.60114	-2.34687	-0.61009
C	2.12545	-0.80863	0.28165
C	2.54815	-1.65445	-0.75298
C	3.06591	-0.36108	1.22387
C	3.88375	-2.04846	-0.81728
C	4.39936	-0.75064	1.14027
C	4.81631	-1.60515	0.12007
H	1.83152	-1.99806	-1.49525

H	2.73597	0.29266	2.03742
H	4.19842	-2.71212	-1.62655
H	5.11204	-0.39285	1.88741
H	5.85930	-1.92285	0.05645
C	-2.79681	-0.06157	0.19023
C	-3.48401	1.15217	0.03121
C	-3.52340	-1.25876	0.21533
C	-4.86707	1.17308	-0.11163
C	-4.90879	-1.22457	0.06069
C	-5.58990	-0.01990	-0.10577
H	-2.91631	2.08774	0.02041
H	-3.00529	-2.20423	0.35493
H	-5.38074	2.12976	-0.23424
H	-5.46343	-2.16604	0.07984
H	-6.67556	-0.00943	-0.22462

Acrolein + 1,3-bis(3,5-bis(trifluoromethyl)phenyl)thiourea (TUF-A)

E = −2547.583708

H = −2547.548708

G = −2547.661712

N_{imag} = 0

O	0.20219	2.90405	0.01650
C	0.51667	4.05735	-0.18509
H	-0.27352	4.82978	-0.32918
C	1.90103	4.54283	-0.25849
C	2.13665	5.84221	-0.47351
H	2.71117	3.81645	-0.13455
H	3.15396	6.23995	-0.53345
H	1.31003	6.55269	-0.59668
H	-0.99808	1.21075	0.06492
H	0.90147	1.10672	0.17248
N	1.01627	0.09644	0.08370
C	-0.14541	-0.63439	0.08368
N	-1.22333	0.21762	0.12343
S	-0.22607	-2.28872	0.04561
C	2.35961	-0.28582	0.04919
C	2.85609	-1.49825	-0.45267
C	3.27188	0.67818	0.50108
C	4.23263	-1.71022	-0.48462
C	4.64126	0.45386	0.43644
C	5.14357	-0.74703	-0.05445
H	2.17371	-2.25915	-0.82530
H	2.89514	1.62110	0.90832
H	6.21820	-0.92466	-0.11221
C	-2.60163	-0.02612	0.10241
C	-3.40711	1.06111	-0.28363
C	-3.22106	-1.22531	0.46957
C	-4.78744	0.94585	-0.31024
C	-4.61370	-1.32063	0.41964
C	-5.41331	-0.25382	0.03347

H	-2.93881	2.00674	-0.56786
H	-2.62666	-2.07606	0.79552
H	-6.50035	-0.34792	0.00600
C	5.55869	1.53928	0.93253
F	5.20241	2.73360	0.43378
F	5.51178	1.65444	2.25999
F	6.82414	1.32249	0.58923
C	4.74740	-3.04013	-0.97915
F	5.96998	-2.92491	-1.50100
F	4.82465	-3.92723	0.01329
F	3.95299	-3.55850	-1.91362
C	-5.24010	-2.64516	0.77972
F	-6.55307	-2.53050	0.98263
F	-5.06280	-3.54355	-0.18895
F	-4.70020	-3.15131	1.88832
C	-5.64458	2.12341	-0.69449
F	-4.91504	3.14496	-1.14893
F	-6.51968	1.79656	-1.64501
F	-6.34896	2.56943	0.34716

TS: endo_CP + A

E = −385.343710

H = −385.334897

G = −385.376146

N_{imag} = 1,-465.2270i cm^{-1}

C	-0.93989	0.85934	0.31751
C	-0.02044	1.62617	-0.38808
H	0.40658	2.52090	0.07372
C	-1.93786	0.04076	-0.38664
O	-2.92096	-0.44529	0.11168
H	-1.70530	-0.11704	-1.47432
H	-1.15302	1.05641	1.37226
H	-0.08099	1.65468	-1.48033
C	0.41253	-0.96327	0.84893
C	0.67006	-1.43195	-0.43610
C	1.53831	-0.53232	-1.07979
C	1.81182	0.53305	-0.21592
C	1.46148	0.06046	1.17240
H	-0.19488	-1.48019	1.59481
H	0.18486	-2.29068	-0.90335
H	1.84259	-0.58722	-2.12687
H	2.57302	1.29220	-0.41138
H	1.17423	0.83759	1.89319
H	2.34460	-0.47773	1.57490

TS: exo_CP + A

E = −385.342776

H = −385.333980

G = −385.375205

N_{imag} = 1,−460.6969i cm^{-1}

C	-2.13339	-0.02150	-0.55796
C	-1.35122	-1.17642	-0.74380
C	-0.37188	-1.21782	0.24116
C	-1.62822	0.70006	0.52839
C	-0.78119	-0.26026	1.32206
H	-2.92182	0.32276	-1.23016
H	-1.42557	-1.86254	-1.58911
H	0.35784	-2.01919	0.37511
H	-2.13608	1.55727	0.97709
H	0.03009	0.18232	1.91565
H	-1.45553	-0.80385	2.01553
C	0.92384	0.51741	-0.67600
C	0.09087	1.59123	-0.38301
C	2.07833	0.20735	0.17911
H	2.09740	0.77285	1.15072
O	2.95716	-0.57275	-0.08837
H	0.95078	0.07381	-1.67355
H	0.32600	2.21249	0.48975
H	-0.44326	2.10586	-1.18369

PC: endo_CP + A

E = −385.405568

H = −385.397453

G = −385.437502

N_{imag} = 0

C	-0.73696	0.50900	0.30359
C	0.17875	1.23460	-0.72383
H	0.16873	2.32190	-0.55178
C	-1.91522	-0.18990	-0.32300
O	-3.05049	-0.07939	0.04873
H	-1.65999	-0.85279	-1.19098
H	-1.13786	1.20526	1.05557
H	-0.13172	1.05890	-1.76699
C	0.26510	-0.48320	1.00226
C	0.73005	-1.47697	-0.04917
C	1.52321	-0.81488	-0.90186
C	1.58805	0.62798	-0.43578
C	1.50336	0.42888	1.08855
H	-0.12287	-0.90418	1.93889
H	0.38944	-2.51108	-0.13371
H	1.95865	-1.19575	-1.82822
H	2.42282	1.22798	-0.82143
H	1.31757	1.36377	1.64355
H	2.38108	-0.09204	1.50070

PC: exo_CP + A
E = −385.405056
H = −385.396891
G = −385.437131
N_{imag} = 0

C	-2.11705	-0.17308	-0.59131
C	-1.41473	-1.29196	-0.37026
C	-0.22938	-0.91815	0.50048
C	-1.40768	0.95868	0.12931
C	-0.85193	0.20553	1.35104
H	-2.97699	-0.05437	-1.25372
H	-1.57464	-2.27535	-0.81691
H	0.26907	-1.74314	1.02761
H	-2.00070	1.86266	0.32138
H	-0.11775	0.79227	1.92896
H	-1.64195	-0.16714	2.02040
C	0.72176	-0.09403	-0.44848
C	-0.08508	1.21955	-0.65560
C	2.06390	0.14033	0.19804
H	2.04119	0.75551	1.13729
O	3.10358	-0.29150	-0.21469
H	0.90889	-0.63850	-1.38467
H	0.44534	2.08586	-0.22243
H	-0.25998	1.43301	-1.71980

TS: endo_CP + U
E = −610.284702
H = −610.269952
G = −610.326211
N_{imag} = 1,−460.6106i cm^{-1}

C	-0.91176	1.22063	-0.71766
C	-2.30622	1.18570	-0.76039
C	-0.25509	1.84509	0.42030
O	0.89253	2.24661	0.46668
H	-0.91442	1.95952	1.31812
C	-0.58311	-1.10186	-0.00712
C	-1.13907	-0.94221	1.25670
C	-2.52733	-0.76201	1.12499
C	-2.85941	-0.73834	-0.23746
C	-1.70090	-1.37246	-0.96542
H	2.10073	1.34618	-0.82028
H	-0.30376	1.03791	-1.60756
H	-2.80434	1.14001	-1.73334
H	-2.86613	1.72910	0.00681
H	0.46213	-1.34364	-0.21100
H	-0.56622	-0.88135	2.18337
H	-3.21856	-0.54658	1.94254
H	-3.88817	-0.79085	-0.60274
H	-1.53608	-1.05020	-2.00270
H	-1.86580	-2.46979	-0.97021
H	2.31497	0.85298	1.39557

N	2.40792	-0.13225	1.16993
C	2.67055	-0.53102	-0.12283
H	2.72078	-0.77224	1.88970
N	2.53130	0.45498	-1.06062
H	2.62228	0.16882	-2.02652
O	2.96272	-1.67913	-0.40089

TS: exo_CP + U-A
E = -610.284689
H = -610.270159
G = -610.325662
$\boldsymbol{N}_{\mathbf{imag}}$ = 1,-438.5839i cm^{-1}

C	-2.36474	1.69462	0.51789
C	-0.96571	1.67867	0.35966
C	-0.63820	0.78051	-0.64562
C	-2.93593	0.75070	-0.34511
C	-1.89459	0.45500	-1.39431
H	-2.90877	2.25908	1.27813
H	-0.24110	2.20152	0.98545
H	0.37416	0.57290	-1.00561
H	-4.00968	0.68252	-0.53738
H	-1.94026	-0.53761	-1.86336
H	-2.00893	1.21305	-2.19627
C	-1.16951	-1.13152	0.77617
C	-2.55690	-1.02656	0.71336
C	-0.43370	-2.00457	-0.11867
H	-1.03877	-2.45249	-0.94759
O	0.75092	-2.27887	-0.03092
H	-0.61451	-0.73640	1.62989
H	-3.09698	-1.66860	0.00712
H	-3.11890	-0.76374	1.61157
H	1.72472	-0.89966	1.11393
H	2.24130	-1.18501	-1.03303
N	2.65511	-0.25663	-1.09079
H	3.31187	-0.07654	-1.84162
C	2.91242	0.44290	0.07379
N	2.16120	0.02100	1.14781
H	2.45594	0.38929	2.04431
O	3.67870	1.38045	0.12249

PC: endo_CP + U-A
E = -610.338571
H = -610.323908
G = -610.383489
$\boldsymbol{N}_{\mathbf{imag}}$ = 0

C	1.29455	-0.49279	0.08806
C	2.32851	-0.72705	1.22965
C	0.25087	0.52620	0.43391
O	-0.93745	0.35319	0.32884
H	0.64175	1.50385	0.80835
C	2.21437	-0.04479	-1.11066

C	2.85394	1.27900	-0.72675
C	3.74072	1.02722	0.24521
C	3.69674	-0.46327	0.52720
C	3.39914	-1.00271	-0.88358
H	-2.55749	-0.96082	-0.12335
H	0.77279	-1.42267	-0.18392
H	2.26071	-1.76048	1.60219
H	2.17625	-0.05289	2.08831
H	1.70802	-0.08362	-2.08375
H	2.56340	2.25633	-1.11787
H	4.31914	1.75750	0.81510
H	4.56013	-0.88499	1.05802
H	3.10922	-2.06673	-0.89552
H	4.22205	-0.82386	-1.59196
H	-2.89013	1.22887	0.39600
N	-3.85280	1.15520	0.08387
C	-4.44392	-0.07587	-0.07918
H	-4.48761	1.92430	0.25466
N	-3.55630	-1.12640	-0.05843
H	-3.93053	-2.01956	-0.35117
O	-5.63925	-0.22227	-0.22838

PC: exo_CP + U-A
E = −610.337904
H = −610.323185
G = −601.383006
N_{imag} = 0

C	-3.95623	1.05467	0.10592
C	-3.02352	1.41864	-0.78367
C	-2.12443	0.21782	-1.01328
C	-3.68955	-0.39162	0.47978
C	-3.14158	-0.92790	-0.85443
H	-4.71010	1.69420	0.56937
H	-2.84872	2.41486	-1.19453
H	-1.50842	0.23569	-1.92267
H	-4.52269	-0.93915	0.93936
H	-2.68630	-1.92949	-0.76963
H	-3.89381	-0.92892	-1.65731
C	-1.29638	0.08137	0.32439
C	-2.38375	-0.39135	1.33311
C	-0.18551	-0.91449	0.16443
H	-0.49959	-1.96585	-0.05348
O	0.98750	-0.64879	0.23718
H	-0.83965	1.04187	0.60148
H	-2.17304	-1.41031	1.70231
H	-2.44717	0.27307	2.20642
H	2.99379	-1.28534	-0.20030
H	2.50344	0.82827	0.48734
N	3.49458	1.03083	0.56358
H	3.79446	1.97901	0.74913
C	4.42546	0.22033	-0.04228

N	3.91352	-0.97405	-0.49454
H	4.60008	-1.66306	-0.77340
O	5.59175	0.53039	-0.16858

TS: endo_CP + UPh-A
E = -1071.688596
H = -1071.665058
G = -1071.744236
N_{imag} = 1,-453.6662i cm^{-1}

C	-0.92225	-1.53658	0.45082
C	-1.37265	-2.24688	1.56251
C	-0.41362	-2.18959	-0.73989
O	0.18909	-1.64323	-1.65230
H	-0.61023	-3.28910	-0.79338
C	-3.07844	-1.27345	-0.63734
C	-3.43307	-2.61325	-0.73957
C	-3.69412	-3.10778	0.55014
C	-3.44824	-2.09426	1.48792
C	-3.46929	-0.79085	0.72542
H	0.00691	0.52631	-1.54901
H	-0.74688	-0.46134	0.53028
H	-1.31328	-1.76889	2.54457
H	-1.25958	-3.33523	1.57925
H	-2.84769	-0.61309	-1.47437
H	-3.41558	-3.20605	-1.65562
H	-3.92255	-4.14701	0.79580
H	-3.74019	-2.16940	2.53833
H	-2.87899	0.04304	1.13230
H	-4.52719	-0.45889	0.66934
H	1.72994	-0.44144	-1.17619
N	2.16622	0.28425	-0.60859
C	1.35575	1.32780	-0.22201
N	0.13190	1.28510	-0.88067
O	1.65342	2.18053	0.58500
C	3.48096	0.02480	-0.19984
C	4.24352	0.89594	0.59279
C	4.05182	-1.18386	-0.63166
C	5.54962	0.54526	0.93351
C	5.35557	-1.51741	-0.28282
C	6.11701	-0.65349	0.50498
H	3.80954	1.83182	0.93644
H	3.45666	-1.86333	-1.24954
H	6.13344	1.23312	1.55076
H	5.77843	-2.46286	-0.63186
H	7.14152	-0.91252	0.78060
C	-1.03983	1.94834	-0.49748
C	-2.11822	1.93063	-1.39669
C	-1.20667	2.57232	0.75006
C	-3.34590	2.48682	-1.04979
C	-2.43940	3.13487	1.07955
C	-3.51784	3.09145	0.19581

H	-1.97864	1.47538	-2.38203
H	-0.36933	2.61931	1.44366
H	-4.17072	2.45661	-1.76653
H	-2.55371	3.61784	2.05355
H	-4.47700	3.53687	0.46821

TS: exo_CP + UPh-A

E = -1071.691932

H = -1071.668590

G = -1071.746104

N_{imag} = 1,-436.4597i cm^{-1}

C	-3.41909	-2.12893	-1.20430
C	-2.76144	-0.96423	-1.12593
C	-1.31293	-1.28596	-0.80335
C	-2.42181	-3.23879	-0.92762
C	-1.14837	-2.62901	-1.53793
H	-4.49429	-2.26999	-1.33187
H	-3.17590	0.04677	-1.16564
H	-0.58319	-0.49234	-1.01883
H	-2.70685	-4.24716	-1.25588
H	-0.22799	-3.17795	-1.27326
H	-1.20944	-2.51128	-2.62998
C	-1.35597	-1.76574	0.70557
C	-2.06942	-3.14434	0.58857
C	0.01803	-1.87882	1.29025
H	0.68311	-2.65773	0.84050
O	0.44144	-1.17954	2.18142
H	-1.91822	-1.04563	1.31659
H	-1.39429	-3.97071	0.87321
H	-2.95696	-3.19963	1.23482
H	1.81632	0.31437	1.64763
H	-0.15513	0.87885	1.76984
N	-0.18504	1.39985	0.89715
C	0.96166	1.38152	0.11866
N	2.02074	0.78247	0.76991
O	1.02296	1.83386	-1.00431
C	3.28078	0.47905	0.22927
C	3.80120	1.08197	-0.92486
C	4.05148	-0.47502	0.91277
C	5.07225	0.72108	-1.37070
C	5.32013	-0.81836	0.45837
C	5.84031	-0.22302	-0.69100
H	3.21021	1.81944	-1.46259
H	3.64082	-0.95058	1.80906
H	5.46771	1.19726	-2.27157
H	5.90372	-1.56141	1.00758
H	6.83524	-0.49202	-1.05204
C	-1.46139	1.82984	0.51095
C	-2.51442	1.58578	1.40979
C	-1.74305	2.46993	-0.70667
C	-3.81940	1.94939	1.09624

C	-3.05752	2.83088	-1.00405
C	-4.10373	2.57228	-0.11945
H	-2.29861	1.10634	2.36988
H	-0.93805	2.67446	-1.40749
H	-4.61908	1.74457	1.81222
H	-3.26101	3.32692	-1.95657
H	-5.12703	2.85984	-0.36945

PC: endo_CP + UPh-A

E = −1071.745927

H = −1071.722943

G = −1071.802435

$\boldsymbol{N}_{\mathbf{imag}}$ = 0

C	-1.77412	-1.38812	0.01310
C	-1.82911	-2.14106	1.37471
C	-0.89847	-2.05024	-1.00483
O	-0.05716	-1.48649	-1.66685
H	-1.06592	-3.14573	-1.14685
C	-3.29145	-1.35348	-0.41472
C	-3.74393	-2.78706	-0.63192
C	-3.79188	-3.37626	0.57038
C	-3.35954	-2.34827	1.59951
C	-3.91815	-1.06275	0.96133
H	0.05464	0.55320	-1.34038
H	-1.42452	-0.35452	0.14904
H	-1.38451	-1.52266	2.16896
H	-1.28790	-3.10122	1.35536
H	-3.49776	-0.63954	-1.22265
H	-3.90632	-3.25792	-1.60387
H	-3.99526	-4.42848	0.78092
H	-3.63334	-2.55622	2.64199
H	-3.53839	-0.13505	1.42119
H	-5.01792	-1.04891	0.92287
H	1.76415	-0.48446	-1.14972
N	2.24594	0.16819	-0.53758
C	1.53349	1.27465	-0.11711
N	0.28523	1.32319	-0.71573
O	1.94301	2.09815	0.67042
C	3.54441	-0.20776	-0.16708
C	4.38180	0.56455	0.65242
C	4.01677	-1.43422	-0.66304
C	5.66093	0.09951	0.95597
C	5.29540	-1.88214	-0.35111
C	6.13016	-1.11712	0.46382
H	4.02640	1.51460	1.04351
H	3.36572	-2.03888	-1.30216
H	6.30289	0.71149	1.59494
H	5.64002	-2.83929	-0.75057
H	7.13503	-1.46592	0.71104
C	-0.77270	2.19155	-0.42562
C	-1.96461	1.99913	-1.14634

C	-0.71996	3.20689	0.54189
C	-3.08422	2.78584	-0.89775
C	-1.85070	3.98916	0.77586
C	-3.03689	3.78850	0.07128
H	-1.99972	1.22360	-1.91886
H	0.20006	3.37221	1.09745
H	-3.99808	2.61608	-1.47312
H	-1.79534	4.77617	1.53245
H	-3.91217	4.41089	0.26846

PC: exo_CP + UPh-A

E = −1071.691932

H = −1071.668590

G = −1070.746104

$\boldsymbol{N}_{\mathbf{imag}}$ = 0

C	-3.41909	-2.12893	-1.20430
C	-2.76144	-0.96423	-1.12593
C	-1.31293	-1.28596	-0.80335
C	-2.42181	-3.23879	-0.92762
C	-1.14837	-2.62901	-1.53793
H	-4.49429	-2.26999	-1.33187
H	-3.17590	0.04677	-1.16564
H	-0.58319	-0.49234	-1.01883
H	-2.70685	-4.24716	-1.25588
H	-0.22799	-3.17795	-1.27326
H	-1.20944	-2.51128	-2.62998
C	-1.35597	-1.76574	0.70557
C	-2.06942	-3.14434	0.58857
C	0.01803	-1.87882	1.29025
H	0.68311	-2.65773	0.84050
O	0.44144	-1.17954	2.18142
H	-1.91822	-1.04563	1.31659
H	-1.39429	-3.97071	0.87321
H	-2.95696	-3.19963	1.23482
H	1.81632	0.31437	1.64763
H	-0.15513	0.87885	1.76984
N	-0.18504	1.39985	0.89715
C	0.96166	1.38152	0.11866
N	2.02074	0.78247	0.76991
O	1.02296	1.83386	-1.00431
C	3.28078	0.47905	0.22927
C	3.80120	1.08197	-0.92486
C	4.05148	-0.47502	0.91277
C	5.07225	0.72108	-1.37070
C	5.32013	-0.81836	0.45837
C	5.84031	-0.22302	-0.69100
H	3.21021	1.81944	-1.46259
H	3.64082	-0.95058	1.80906
H	5.46771	1.19726	-2.27157
H	5.90372	-1.56141	1.00758
H	6.83524	-0.49202	-1.05204

C	-1.46139	1.82984	0.51095
C	-2.51442	1.58578	1.40979
C	-1.74305	2.46993	-0.70667
C	-3.81940	1.94939	1.09624
C	-3.05752	2.83088	-1.00405
C	-4.10373	2.57228	-0.11945
H	-2.29861	1.10634	2.36988
H	-0.93805	2.67446	-1.40749
H	-4.61908	1.74457	1.81222
H	-3.26101	3.32692	-1.95657
H	-5.12703	2.85984	-0.36945

TS: endo_CP + UF-A

E = -2418.440410

H = -2418.403313

G = -2418.517151

$\boldsymbol{N}_{\mathbf{imag}}$ = 1,-444.7589i cm^{-1}

C	-1.52576	-2.53082	1.08387
C	-1.69065	-3.19287	2.30328
C	-0.67638	-3.08168	0.05367
O	-0.24418	-2.48284	-0.92483
H	-0.39790	-4.15355	0.19749
C	-3.60590	-3.41411	0.02728
C	-3.25566	-4.75647	0.07223
C	-3.25428	-5.17742	1.41435
C	-3.53828	-4.08017	2.24113
C	-4.17266	-3.03411	1.35867
H	-0.63660	-0.66782	-0.94802
H	-1.84279	-1.48912	0.97255
H	-1.93382	-2.59689	3.18794
H	-1.07176	-4.07096	2.51069
H	-3.74545	-2.83217	-0.88658
H	-2.95567	-5.36198	-0.78455
H	-2.96059	-6.16859	1.76619
H	-3.78243	-4.18365	3.30127
H	-4.04540	-1.98541	1.65811
H	-5.26252	-3.23913	1.32068
H	1.31650	-1.04845	-0.86380
N	1.62796	-0.12105	-0.57885
C	0.64838	0.84171	-0.40755
N	-0.60025	0.32753	-0.70170
O	0.85554	1.97878	-0.05008
C	2.99890	0.02462	-0.37160
C	3.61195	1.22307	0.01725
C	3.79865	-1.11461	-0.56921
C	4.99460	1.25360	0.20359
C	5.17156	-1.05335	-0.38229
C	5.79223	0.13275	0.01133
H	3.01206	2.11803	0.17365
H	3.33451	-2.05782	-0.86871
H	6.87102	0.17759	0.16644

C	-1.82771	0.96279	-0.57416
C	-2.96024	0.20490	-0.91841
C	-2.00825	2.27867	-0.12266
C	-4.23547	0.73718	-0.79419
C	-3.30106	2.79244	-0.02060
C	-4.42784	2.04290	-0.34444
H	-2.82305	-0.81084	-1.29972
H	-1.14377	2.88593	0.14260
H	-5.42872	2.46668	-0.25677
C	6.01862	-2.27018	-0.65068
F	6.56398	-2.22435	-1.86771
F	7.02032	-2.36884	0.22339
F	5.30585	-3.39639	-0.58067
C	5.60743	2.55585	0.65458
F	6.93982	2.50377	0.65456
F	5.24165	3.56493	-0.13580
F	5.21908	2.86872	1.89201
C	-5.41806	-0.14056	-1.10209
F	-6.48521	0.56154	-1.46544
F	-5.14537	-1.01246	-2.07818
F	-5.77243	-0.87096	-0.03236
C	-3.45064	4.22043	0.44415
F	-4.72823	4.54198	0.65150
F	-2.79112	4.43017	1.58333
F	-2.96268	5.07499	-0.45479

TS: exo_CP + UF-A
E = −2418.441687
H = −2418.403739
G = −2418.520680
N_{imag} = 1,−433.6023i cm^{-1}

C	3.70378	-2.07810	2.53218
C	2.97649	-1.05341	1.89417
C	1.63588	-1.40304	1.88415
C	2.83236	-3.12003	2.87786
C	1.44213	-2.53513	2.84458
H	4.79042	-2.10625	2.63462
H	3.40807	-0.16676	1.42462
H	0.82357	-0.79951	1.47092
H	3.11469	-3.93477	3.54940
H	0.62260	-3.23206	2.62241
H	1.24794	-2.09408	3.84388
C	2.03467	-3.22500	0.19536
C	2.65566	-4.11154	1.07627
C	0.62389	-3.31001	-0.08885
H	0.05361	-4.04299	0.53211
O	0.02220	-2.66698	-0.94220
H	2.63832	-2.57903	-0.44487
H	2.06151	-4.93708	1.48671
H	3.71466	-4.33896	0.93824
H	-1.33900	-1.21826	-0.97078

H	0.61085	-0.91629	-1.37730
N	0.64678	0.07849	-1.13628
C	-0.55029	0.66958	-0.76480
N	-1.58631	-0.24421	-0.79216
O	-0.66463	1.83264	-0.45077
C	-2.93315	0.00455	-0.52790
C	-3.46278	1.27405	-0.26471
C	-3.79693	-1.10532	-0.53423
C	-4.82977	1.40522	-0.01369
C	-5.15105	-0.94471	-0.28392
C	-5.68971	0.31501	-0.01622
H	-2.81192	2.14673	-0.25208
H	-3.39615	-2.10231	-0.73449
H	-6.75418	0.43877	0.18803
C	1.92182	0.60471	-0.96785
C	2.99063	-0.15071	-1.47136
C	2.20255	1.79883	-0.28066
C	4.30521	0.22931	-1.22812
C	3.52851	2.16831	-0.07400
C	4.59733	1.39524	-0.52826
H	2.78552	-1.05805	-2.04651
H	1.38846	2.41890	0.09079
H	5.62907	1.69862	-0.34504
C	-6.07373	-2.13461	-0.33849
F	-6.75671	-2.16841	-1.48391
F	-5.40610	-3.28593	-0.24116
F	-6.96896	-2.09995	0.64913
C	-5.35159	2.78879	0.28345
F	-6.67772	2.80144	0.41889
F	-4.82810	3.27104	1.41178
F	-5.03616	3.64428	-0.68922
C	3.82416	3.38839	0.76168
F	3.98985	3.04967	2.04511
F	4.94716	3.99028	0.36933
F	2.84018	4.28061	0.71105
C	5.40025	-0.70610	-1.66508
F	6.60408	-0.14893	-1.57775
F	5.41135	-1.80400	-0.89214
F	5.22672	-1.12204	-2.91861

PC: endo_CP + UF-A
E = −2418.469001
H = −2418.458745
G = −2418.573998
N_{imag} = 0

C	-2.00634	2.72940	-0.70540
C	-1.98143	3.25743	-2.17121
C	-0.88693	3.25535	0.13614
O	-0.17548	2.57445	0.84204
H	-0.72330	4.35827	0.09535
C	-3.42880	3.19801	-0.20425

C	-3.43592	4.71705	-0.20426
C	-3.43029	5.11599	-1.48301
C	-3.40538	3.87021	-2.34886
C	-4.23843	2.90929	-1.48148
H	-0.62168	0.66479	0.96255
H	-1.96353	1.62923	-0.68546
H	-1.81055	2.42571	-2.87116
H	-1.19112	4.00650	-2.34228
H	-3.75987	2.70873	0.72166
H	-3.37119	5.34734	0.68517
H	-3.35333	6.14043	-1.85350
H	-3.70686	3.99738	-3.39655
H	-4.18256	1.85585	-1.80232
H	-5.28986	3.21665	-1.38090
H	1.34062	1.07257	0.82680
N	1.65045	0.14028	0.56212
C	0.67487	-0.82917	0.41138
N	-0.57773	-0.32337	0.71194
O	0.88480	-1.96815	0.06470
C	3.02288	-0.00206	0.35139
C	3.63889	-1.20870	-0.01713
C	3.81574	1.14310	0.51446
C	5.01776	-1.23765	-0.20904
C	5.19034	1.08479	0.31692
C	5.81209	-0.10451	-0.05186
H	3.04079	-2.10614	-0.16071
H	3.34910	2.09328	0.78941
H	6.88853	-0.14459	-0.22458
C	-1.80339	-0.97216	0.59313
C	-2.93850	-0.24944	0.99809
C	-1.97375	-2.26857	0.08849
C	-4.20922	-0.78907	0.86361
C	-3.26190	-2.79464	-0.01714
C	-4.39198	-2.07482	0.35517
H	-2.81508	0.74542	1.43463
H	-1.10681	-2.85418	-0.21463
H	-5.38890	-2.50698	0.26262
C	6.00981	2.32813	0.54820
F	7.16717	2.28554	-0.10873
F	5.35588	3.42358	0.15294
F	6.29274	2.49079	1.84218
C	5.67575	-2.55069	-0.55501
F	4.85619	-3.35201	-1.23229
F	6.77033	-2.36589	-1.29522
F	6.05225	-3.20677	0.54456
C	-5.40151	0.05841	1.22059
F	-6.43494	-0.67654	1.61848
F	-5.11486	0.92819	2.19396
F	-5.81193	0.78160	0.17000
C	-3.39685	-4.19247	-0.56986
F	-4.66563	-4.60107	-0.58089

F	-2.94082	-4.26062	-1.82135
F	-2.69561	-5.06660	0.15051

PC: exo_CP + UF-A

E = -2418.496735

H = -2418.459436

G = -2418.575471

N_{imag} = 0

C	3.92482	1.97032	-2.43273
C	3.12250	1.03726	-1.90357
C	1.74748	1.66158	-1.75557
C	3.09610	3.22366	-2.64618
C	1.72847	2.59868	-2.97788
H	5.00132	1.89411	-2.59713
H	3.39715	0.03840	-1.55598
H	0.90514	0.96727	-1.62179
H	3.49904	3.95902	-3.35460
H	0.90007	3.32859	-2.97468
H	1.73041	2.04123	-3.92611
C	1.90495	2.71825	-0.58010
C	2.79949	3.80560	-1.22972
C	0.55101	3.19710	-0.15603
H	0.06659	3.97273	-0.79735
O	-0.05309	2.76174	0.79928
H	2.38402	2.24219	0.28516
H	2.26483	4.76774	-1.31515
H	3.71496	3.97734	-0.64599
H	-1.39728	1.23033	1.07056
H	0.58658	0.93037	1.43076
N	0.62041	-0.04963	1.15186
C	-0.57741	-0.63329	0.76882
N	-1.62619	0.26270	0.85422
O	-0.68409	-1.78169	0.40641
C	-2.97130	0.01149	0.57355
C	-3.48723	-1.25500	0.26946
C	-3.84569	1.11006	0.61852
C	-4.85279	-1.39286	0.01924
C	-5.19964	0.94362	0.36484
C	-5.72574	-0.31260	0.06408
H	-2.82851	-2.12086	0.23271
H	-3.45831	2.10308	0.86201
H	-6.79250	-0.44445	-0.12158
C	1.89780	-0.57996	0.98441
C	2.96589	0.15274	1.52042
C	2.17733	-1.76025	0.27534
C	4.28074	-0.23190	1.28609
C	3.50319	-2.13835	0.08151
C	4.57202	-1.38496	0.56602
H	2.76489	1.04717	2.11736
H	1.36416	-2.36440	-0.12314
H	5.60347	-1.69186	0.38832

C	-6.10819	2.14599	0.36445
F	-7.32670	1.83802	0.80559
F	-5.62993	3.12563	1.13392
F	-6.24955	2.64655	-0.86508
C	-5.36350	-2.76823	-0.33231
F	-4.90412	-3.68828	0.51538
F	-6.69517	-2.82289	-0.30915
F	-4.96941	-3.13061	-1.55429
C	3.79029	-3.34025	-0.78300
F	4.94968	-3.91264	-0.46002
F	2.83628	-4.26161	-0.68893
F	3.87258	-2.98292	-2.06931
C	5.37483	0.68069	1.77315
F	6.57863	0.12915	1.65701
F	5.38422	1.82050	1.06910
F	5.19783	1.02063	3.05041

TS: endo_CP + TU-A
E = −933.189032
H = −993.174058
G = −993.230977
$\boldsymbol{N_{imag}}$ = 1,−457.0710i cm^{-1}

C	1.38030	-1.12111	-0.81266
C	2.74234	-0.82342	-0.89472
C	0.89067	-1.92230	0.29259
O	-0.18391	-2.49554	0.35669
H	1.60551	-2.00256	1.15004
C	0.67582	1.05522	0.13302
C	1.33331	0.89658	1.34739
C	2.72024	0.97506	1.13037
C	2.96270	1.11435	-0.24477
C	1.66653	1.58677	-0.85457
H	-1.57769	-1.77851	-0.85886
H	0.70891	-0.97451	-1.66277
H	3.17718	-0.61873	-1.87759
H	3.42625	-1.31442	-0.19569
H	-0.40772	1.12461	-0.00299
H	0.84623	0.66992	2.29737
H	3.48983	0.82636	1.89082
H	3.94047	1.38467	-0.65162
H	1.48969	1.32100	-1.90564
H	1.63969	2.69320	-0.77380
H	-1.71148	-1.48337	1.37331
N	-2.14858	-0.57495	1.23472
C	-2.48397	-0.13298	0.00125
H	-2.45328	-0.01791	2.02239
N	-2.14680	-0.94629	-1.02059
H	-2.36787	-0.62863	-1.95515
S	-3.22465	1.35268	-0.23686

TS: exo_CP + TU-A

E = −933.189032
H = −993.174058
G = −993.230977
N_{imag} = 1,−457.0710i cm^{-1}

C	-2.09395	1.90203	0.55844
C	-0.74598	1.65955	0.22871
C	-0.70293	0.72365	-0.79345
C	-2.91764	1.06543	-0.20815
C	-2.07811	0.59983	-1.37134
H	-2.43984	2.55246	1.36461
H	0.13985	2.06424	0.72446
H	0.21030	0.36763	-1.27515
H	-4.00186	1.18860	-0.27278
H	-2.34026	-0.37490	-1.80579
H	-2.17107	1.36285	-2.17128
C	-1.40759	-1.09303	0.75822
C	-2.75348	-0.73496	0.81527
C	-0.92303	-2.09917	-0.16429
H	-1.65552	-2.41126	-0.95156
O	0.17916	-2.62374	-0.13804
H	-0.71965	-0.78747	1.54901
H	-3.46261	-1.27559	0.17664
H	-3.17282	-0.38378	1.76024
H	1.92841	-1.81392	-1.05123
H	1.43032	-1.63140	1.12354
N	1.93837	-0.74884	1.19519
H	1.91033	-0.19854	2.04380
C	2.39777	-0.14008	0.08191
N	2.36052	-0.89230	-1.04218
H	2.71669	-0.47635	-1.89290
S	2.96574	1.43724	0.07998

PC: endo_CP + TU-A

E = −933.241919
H = −933.226980
G = −933.286917
N_{imag} = 0

C	1.77608	-0.46386	0.21310
C	2.83883	-0.44815	1.35245
C	0.74514	0.61379	0.35100
O	-0.44714	0.43185	0.31366
H	1.14613	1.64660	0.49206
C	2.66558	-0.29565	-1.07768
C	3.31587	1.07606	-1.01007
C	4.22649	1.03832	-0.02828
C	4.18855	-0.35346	0.57539
C	3.85472	-1.18793	-0.67447
H	-2.04715	-0.90215	0.06513
H	1.24664	-1.42732	0.16502
H	2.77943	-1.37454	1.94368

H	2.70905	0.39844	2.04625
H	2.13443	-0.54448	-2.00547
H	3.01751	1.94502	-1.60040
H	4.82072	1.87188	0.35214
H	5.06482	-0.65352	1.16453
H	3.56405	-2.22681	-0.44519
H	4.65918	-1.17407	-1.42512
H	-2.35209	1.32156	0.30935
N	-3.35642	1.20815	0.20017
C	-3.91780	-0.00534	0.00068
H	-3.97812	2.00453	0.23572
N	-3.04882	-1.04074	-0.03769
H	-3.43561	-1.96223	-0.19009
S	-5.56724	-0.21202	-0.18544

PC: exo_CP + TU-A
E = −933.241150
H = −933.226153
G = −933.286543
$\boldsymbol{N}_{\mathbf{imag}}$ = 0

C	-4.42651	1.08667	0.24288
C	-3.59254	1.41113	-0.75364
C	-2.73021	0.19451	-1.03547
C	-4.13035	-0.34865	0.63623
C	-3.73096	-0.93540	-0.72905
H	-5.12314	1.74877	0.76093
H	-3.45753	2.39079	-1.21600
H	-2.21557	0.17537	-2.00588
H	-4.91275	-0.87222	1.20081
H	-3.27573	-1.93813	-0.65814
H	-4.56432	-0.95668	-1.44695
C	-1.76504	0.09805	0.21029
C	-2.74120	-0.33360	1.34518
C	-0.68271	-0.91177	-0.02542
H	-1.02418	-1.95899	-0.21727
O	0.49740	-0.66272	-0.03446
H	-1.27930	1.06414	0.40697
H	-2.49862	-1.34245	1.72244
H	-2.70544	0.35859	2.19818
H	2.46382	-1.37621	-0.27425
H	2.00193	0.77385	0.23862
N	2.99364	0.99589	0.25593
H	3.31581	1.93148	0.46269
C	3.93634	0.06057	0.00119
N	3.46027	-1.17542	-0.27009
H	4.13856	-1.89950	-0.46454
S	5.57244	0.40817	0.01963

TS: endo_CP + TUPh-A

E = −1394.582257

H = −1394.558425

G = −1394.637765

N_{imag} = 1,−454.6219i cm^{-1}

C	-1.35946	-1.61120	0.71717
C	-2.14800	-2.16219	1.72648
C	-0.68690	-2.42918	-0.27331
O	0.16525	-2.04512	-1.06044
H	-1.00611	-3.50050	-0.29331
C	-3.14481	-1.19185	-0.87297
C	-3.62638	-2.48993	-0.99814
C	-4.24656	-2.85603	0.20880
C	-4.11083	-1.80699	1.13016
C	-3.78904	-0.56975	0.32683
H	0.00283	0.18604	-1.26424
H	-1.05673	-0.56208	0.77225
H	-2.26434	-1.60390	2.65993
H	-2.18099	-3.25001	1.84045
H	-2.64333	-0.62527	-1.65951
H	-3.46279	-3.14750	-1.85359
H	-4.65259	-3.84354	0.43788
H	-4.65326	-1.77149	2.07818
H	-3.21582	0.22302	0.82877
H	-4.75326	-0.13001	-0.00424
H	1.61541	-0.78317	-0.75364
N	2.16823	0.01728	-0.43529
C	1.44031	1.13945	-0.17272
N	0.16414	1.00550	-0.67891
S	1.96717	2.49866	0.62540
C	3.51589	-0.30080	-0.19074
C	4.54398	0.63525	-0.01816
C	3.82803	-1.67055	-0.17308
C	5.85005	0.19039	0.18578
C	5.13535	-2.09880	0.02575
C	6.15809	-1.16833	0.21371
H	4.32033	1.69874	-0.04208
H	3.02579	-2.39972	-0.32254
H	6.64197	0.93153	0.32048
H	5.35389	-3.16947	0.03632
H	7.18593	-1.50051	0.37534
C	-0.97656	1.78802	-0.42307
C	-1.90277	1.95619	-1.46016
C	-1.26273	2.32158	0.84224
C	-3.10399	2.62730	-1.23792
C	-2.45667	3.00877	1.04976
C	-3.38581	3.16176	0.01862
H	-1.66595	1.55975	-2.45187
H	-0.54282	2.19819	1.65084
H	-3.81437	2.74696	-2.05976
H	-2.66488	3.42575	2.03831

H	-4.31940	3.70151	0.19218

TS: exo_CP + TUPh-A
E = −1394.584275
H = −1394.560571
G = −1394.639468
N_{imag} = 1,-436.1877i cm^{-1}

C	-4.27604	-1.39970	-1.08854
C	-3.25893	-0.42798	-1.00880
C	-2.03274	-1.05985	-1.14881
C	-3.69745	-2.66981	-1.20836
C	-2.27815	-2.44441	-1.66533
H	-5.34245	-1.20660	-0.95471
H	-3.40254	0.63151	-0.78351
H	-1.06026	-0.56037	-1.14802
H	-4.26706	-3.56660	-1.46528
H	-1.54426	-3.21048	-1.37978
H	-2.29440	-2.38983	-2.77322
C	-2.20806	-2.09247	1.10282
C	-3.18109	-3.03643	0.77426
C	-0.80649	-2.44406	1.10364
H	-0.56830	-3.45860	0.69845
O	0.10625	-1.74156	1.51330
H	-2.47591	-1.14231	1.57221
H	-2.85874	-4.06871	0.59132
H	-4.18394	-2.94409	1.19547
H	1.64410	-0.68090	0.90881
H	-0.03630	0.19731	1.24884
N	0.08571	0.99755	0.62318
C	1.34977	1.15352	0.10264
N	2.13423	0.08934	0.45054
S	1.83418	2.45402	-0.81443
C	3.47637	-0.21683	0.16108
C	4.47197	0.72327	-0.13581
C	3.82252	-1.57678	0.23376
C	5.77669	0.29065	-0.37251
C	5.12858	-1.99278	0.00343
C	6.11706	-1.05913	-0.30972
H	4.22434	1.78059	-0.17940
H	3.04675	-2.30907	0.47805
H	6.54234	1.03526	-0.60466
H	5.37292	-3.05610	0.06594
H	7.14368	-1.38137	-0.49708
C	-1.06722	1.78633	0.49174
C	-1.99375	1.72189	1.54597
C	-1.37962	2.55346	-0.64004
C	-3.20571	2.40192	1.47434
C	-2.59119	3.24205	-0.69390
C	-3.51199	3.17276	0.35201
H	-1.74440	1.13586	2.43576
H	-0.67577	2.60599	-1.46752

H	-3.90838	2.33810	2.30885
H	-2.81869	3.83849	-1.58104
H	-4.45652	3.71827	0.29572

PC: endo_CP + TUPh-A
E = −1394.638937
H = −1394.615562
G = −1394.695157
N_{imag} = 0

C	1.79932	-1.50893	-0.33852
C	2.58512	-1.95213	-1.59892
C	0.83130	-2.50286	0.22567
O	-0.19072	-2.20115	0.79682
H	1.11570	-3.57987	0.13752
C	2.95469	-1.20324	0.69420
C	3.68249	-2.50891	0.96481
C	4.35888	-2.82443	-0.14769
C	4.07450	-1.74477	-1.17549
C	3.94342	-0.51127	-0.25927
H	-0.05475	0.21291	1.29621
H	1.24844	-0.57549	-0.52175
H	2.32251	-1.31493	-2.45706
H	2.38435	-2.99849	-1.88225
H	2.61258	-0.63565	1.56932
H	3.58522	-3.10995	1.87156
H	4.92347	-3.73982	-0.33811
H	4.77473	-1.67687	-2.01810
H	3.51333	0.37624	-0.75536
H	4.88835	-0.24837	0.24062
H	-1.64564	-0.83628	0.71288
N	-2.21059	-0.03159	0.43767
C	-1.49859	1.09994	0.15958
N	-0.21926	0.99125	0.66142
S	-2.05404	2.43523	-0.65515
C	-3.55778	-0.35618	0.19112
C	-4.59751	0.57932	0.11798
C	-3.85440	-1.72284	0.07059
C	-5.90334	0.13844	-0.09263
C	-5.16261	-2.14890	-0.13178
C	-6.19759	-1.21795	-0.22214
H	-4.38265	1.64007	0.22335
H	-3.04120	-2.45182	0.14055
H	-6.70570	0.87822	-0.15033
H	-5.37149	-3.21756	-0.22357
H	-7.22553	-1.54804	-0.38726
C	0.90558	1.80379	0.41710
C	1.81548	1.99736	1.46357
C	1.18765	2.34387	-0.84530
C	2.99472	2.71025	1.25501
C	2.35945	3.07169	-1.03916
C	3.26994	3.25737	0.00282

H	1.58603	1.58788	2.45167
H	0.48196	2.19613	-1.66216
H	3.69331	2.85001	2.08351
H	2.56513	3.49440	-2.02571
H	4.18702	3.82758	-0.16060

PC: exo_CP + TUPh-A

E = -1394.640778

H = -1394.617708

G = -1394.695413

N_{imag} = 0

C	-4.56703	-1.21432	-0.70490
C	-3.53579	-0.36294	-0.78810
C	-2.27039	-1.19645	-0.86259
C	-4.00216	-2.62260	-0.71203
C	-2.78365	-2.42829	-1.63167
H	-5.61761	-0.96126	-0.54896
H	-3.55450	0.72690	-0.70944
H	-1.37114	-0.68511	-1.23396
H	-4.70180	-3.42998	-0.96532
H	-2.08903	-3.28635	-1.62816
H	-3.06400	-2.18158	-2.66643
C	-2.11033	-1.80034	0.59474
C	-3.28588	-2.81324	0.66109
C	-0.76393	-2.44082	0.73737
H	-0.62597	-3.42804	0.23194
O	0.16824	-1.94289	1.32450
H	-2.18500	-0.99727	1.34307
H	-2.91573	-3.84906	0.75843
H	-3.95072	-2.61453	1.51354
H	1.72594	-0.74392	0.92511
H	0.02869	0.16373	1.25430
N	0.14670	0.92239	0.58340
C	1.41740	1.06984	0.07342
N	2.21007	0.02742	0.46755
S	1.90334	2.33914	-0.88180
C	3.55717	-0.26904	0.18076
C	4.55025	0.69093	-0.05042
C	3.90742	-1.62828	0.19296
C	5.86165	0.27874	-0.28439
C	5.22101	-2.02520	-0.03209
C	6.20811	-1.07138	-0.28061
H	4.29484	1.74768	-0.04554
H	3.13073	-2.37545	0.38308
H	6.62725	1.03730	-0.46548
H	5.47151	-3.08875	-0.01810
H	7.23999	-1.37850	-0.46443
C	-0.98560	1.74615	0.45431
C	-1.87017	1.78606	1.54329
C	-1.30449	2.46210	-0.70812
C	-3.04624	2.52854	1.47996

C	-2.47650	3.21576	-0.75388
C	-3.35325	3.25706	0.33112
H	-1.61700	1.23815	2.45613
H	-0.63354	2.42846	-1.56363
H	-3.71808	2.54631	2.34144
H	-2.70912	3.77430	-1.66393
H	-4.26801	3.85157	0.28102

TS: endo_CP + TUF-A

E = -2741.331283

H = -2741.292980

G = -2741.410154

N_{imag} = 1,-449.0877i cm^{-1}

C	0.62944	2.23628	0.54856
C	0.62719	3.12454	1.62829
C	0.41229	2.71716	-0.79888
O	0.08097	2.04596	-1.76879
H	0.55646	3.81788	-0.92719
C	3.10101	2.17762	0.27424
C	3.27302	3.52486	-0.01383
C	3.00030	4.27314	1.14524
C	2.58853	3.40491	2.16743
C	3.04330	2.02438	1.76241
H	0.58227	0.05184	-1.78788
H	0.56593	1.15683	0.71907
H	0.34106	2.74229	2.61272
H	0.33766	4.16446	1.44997
H	3.28586	1.35684	-0.42282
H	3.50119	3.93703	-0.99807
H	2.99127	5.36339	1.20706
H	2.50399	3.71435	3.21206
H	2.45052	1.18270	2.14532
H	4.08498	1.89061	2.12287
H	-1.22450	0.61323	-1.35699
N	-1.53842	-0.25739	-0.91728
C	-0.52878	-1.09367	-0.52166
N	0.65194	-0.71716	-1.11860
S	-0.67504	-2.37445	0.52017
C	-2.90100	-0.28546	-0.59382
C	-3.62736	-1.46126	-0.36238
C	-3.56661	0.94607	-0.55416
C	-4.98674	-1.37851	-0.07434
C	-4.92967	1.00249	-0.28236
C	-5.65586	-0.15712	-0.02959
H	-3.13515	-2.43055	-0.41073
H	-3.00862	1.86953	-0.73345
H	-6.72307	-0.11149	0.19268
C	1.96546	-0.99876	-0.72593
C	2.95970	-0.92111	-1.70619
C	2.33887	-1.21504	0.60942
C	4.30479	-1.01771	-1.35385

C	3.68535	-1.30892	0.93737
C	4.68608	-1.21085	-0.03153
H	2.67707	-0.76184	-2.74990
H	1.57687	-1.27463	1.38572
H	5.74094	-1.27412	0.24269
C	-5.74462	-2.64508	0.23681
F	-7.03201	-2.53587	-0.09599
F	-5.24241	-3.69212	-0.41575
F	-5.69840	-2.93011	1.53899
C	-5.62030	2.34126	-0.30639
F	-5.92331	2.71069	-1.55244
F	-6.75527	2.32383	0.38977
F	-4.84248	3.29935	0.20414
C	5.34690	-0.80374	-2.42042
F	4.99253	-1.36810	-3.57361
F	5.51484	0.50170	-2.65873
F	6.52986	-1.29805	-2.06488
C	4.09580	-1.48553	2.37738
F	4.52972	-2.71804	2.62412
F	5.09161	-0.64807	2.69092
F	3.08759	-1.23436	3.21398

TS: exo_CP + TUF-A

E = −2741.333035

H = −2741.294756

G = −2741.412138

N_{imag} = 1,−432.9042i cm^{-1}

C	4.12686	2.52319	-1.98704
C	3.35552	1.38864	-1.66505
C	2.01505	1.71365	-1.79213
C	3.27433	3.60149	-2.26071
C	1.91782	3.00474	-2.54423
H	5.21492	2.58272	-1.91898
H	3.74859	0.43460	-1.30782
H	1.17740	1.03197	-1.62171
H	3.61847	4.53148	-2.72023
H	1.04817	3.63803	-2.32226
H	1.88800	2.75873	-3.62562
C	2.09206	3.18992	0.24380
C	2.80657	4.23052	-0.35174
C	0.65337	3.21202	0.31423
H	0.15817	4.05114	-0.23136
O	-0.04881	2.40917	0.92024
H	2.61318	2.43608	0.83741
H	2.25229	5.11639	-0.68475
H	3.82554	4.43259	-0.01539
H	-1.32892	0.94329	0.75471
H	0.51638	0.62171	1.11237
N	0.57749	-0.34229	0.76531
C	-0.62349	-0.93456	0.44950
N	-1.63414	-0.01742	0.58094

S	-0.79933	-2.51755	-0.01090
C	-3.01564	-0.12389	0.38552
C	-3.74919	-1.31688	0.39937
C	-3.70013	1.08957	0.20237
C	-5.12979	-1.27325	0.20591
C	-5.07684	1.10723	0.02794
C	-5.81248	-0.07676	0.01749
H	-3.25018	-2.26957	0.56159
H	-3.13973	2.02876	0.19572
H	-6.89333	-0.06412	-0.13032
C	1.88813	-0.80731	0.66122
C	2.83626	-0.15692	1.46383
C	2.32847	-1.79186	-0.23857
C	4.19106	-0.44482	1.34180
C	3.68663	-2.08567	-0.31733
C	4.63693	-1.42326	0.46013
H	2.50427	0.59377	2.18676
H	1.61538	-2.31334	-0.87397
H	5.69831	-1.66223	0.37454
C	-5.78680	2.42961	-0.10303
F	-5.00539	3.35277	-0.66889
F	-6.89049	2.32232	-0.84152
F	-6.15377	2.90594	1.08822
C	-5.88346	-2.57933	0.16116
F	-5.41060	-3.44764	1.05422
F	-7.18229	-2.40566	0.40999
F	-5.78391	-3.15445	-1.03826
C	4.16896	-3.06139	-1.36100
F	5.21527	-3.76388	-0.92714
F	3.22038	-3.91674	-1.72638
F	4.56516	-2.41003	-2.46130
C	5.16812	0.39383	2.12077
F	6.38238	-0.14645	2.15155
F	5.28643	1.60916	1.56239
F	4.76787	0.58605	3.37629

PC: endo_CP + TUF-A
E = -2741.386038
H = -2741.348555
G = -2741.463184
N_{imag} = 0

C	-2.04398	2.60318	-0.75085
C	-2.26364	3.10670	-2.20789
C	-0.78392	3.10526	-0.12198
O	0.00375	2.41304	0.48560
H	-0.58500	4.19775	-0.22560
C	-3.34362	3.11944	-0.01603
C	-3.31195	4.63782	-0.04631
C	-3.52089	5.01561	-1.31439
C	-3.68078	3.75620	-2.14501
C	-4.37245	2.83386	-1.12503

H	-0.49947	0.50117	0.67113
H	-2.03003	1.50281	-0.71528
H	-2.23957	2.25913	-2.90946
H	-1.49728	3.83009	-2.53112
H	-3.51444	2.65296	0.96352
H	-3.07328	5.27990	0.80431
H	-3.48205	6.03124	-1.71420
H	-4.15895	3.87577	-3.12584
H	-4.40029	1.77386	-1.42947
H	-5.38161	3.17248	-0.84743
H	1.33803	0.84459	0.48318
N	1.68952	-0.10338	0.35092
C	0.72079	-1.07078	0.25541
N	-0.51510	-0.49460	0.44133
S	0.97564	-2.68354	-0.01802
C	3.08355	-0.13822	0.23020
C	3.87683	-1.29169	0.15047
C	3.71321	1.11683	0.19985
C	5.25959	-1.16192	0.03543
C	5.09329	1.21811	0.08979
C	5.88855	0.07886	-0.00177
H	3.41904	-2.27790	0.16826
H	3.10819	2.02716	0.25358
H	6.97167	0.15814	-0.10766
C	-1.80560	-1.03070	0.42117
C	-2.78166	-0.28925	1.10792
C	-2.19784	-2.18271	-0.27280
C	-4.11486	-0.67313	1.08264
C	-3.54045	-2.55963	-0.26619
C	-4.51297	-1.82301	0.40306
H	-2.48371	0.59464	1.67847
H	-1.46210	-2.77786	-0.80987
H	-5.55723	-2.14227	0.40217
C	5.72464	2.58573	0.11529
F	6.92544	2.58620	-0.45908
F	4.96729	3.48439	-0.51866
F	5.88182	3.02299	1.36662
C	6.10613	-2.41077	0.00507
F	5.44742	-3.44503	-0.51190
F	7.21339	-2.22873	-0.71627
F	6.49217	-2.76081	1.23427
C	-5.15287	0.17908	1.76376
F	-6.05048	-0.55818	2.41031
F	-4.60412	1.02313	2.64261
F	-5.82250	0.92403	0.87555
C	-3.96286	-3.76311	-1.07329
F	-5.02137	-4.36493	-0.52955
F	-4.30828	-3.40747	-2.31392
F	-2.98638	-4.65960	-1.17575

PC: exo_CP + TUF-A

E = −2741.388007

H = −2741.350284

G = −2741.467823

N_{imag} = 0

C	4.47262	2.54562	-1.57662
C	3.60630	1.52768	-1.49005
C	2.21425	2.12717	-1.43676
C	3.66995	3.83361	-1.58368
C	2.40537	3.36790	-2.32939
H	5.56226	2.48515	-1.54992
H	3.83251	0.46361	-1.39129
H	1.37951	1.44741	-1.66066
H	4.18228	4.72470	-1.96888
H	1.57455	4.09345	-2.27993
H	2.60148	3.09709	-3.37728
C	2.11841	2.81103	-0.00615
C	3.09724	4.00544	-0.14291
C	0.69595	3.19707	0.25715
H	0.33508	4.14659	-0.20640
O	-0.08189	2.52029	0.89267
H	2.42904	2.09693	0.76853
H	2.56851	4.97037	-0.05273
H	3.88152	3.97597	0.62667
H	-1.37686	0.96798	0.81882
H	0.49149	0.59230	1.14915
N	0.53400	-0.33438	0.72121
C	-0.67882	-0.89045	0.38461
N	-1.68253	0.01875	0.60367
S	-0.87889	-2.43005	-0.19163
C	-3.06616	-0.08011	0.40096
C	-3.79832	-1.27228	0.45450
C	-3.74811	1.12764	0.18922
C	-5.17988	-1.23460	0.27139
C	-5.12651	1.14068	0.01954
C	-5.86226	-0.04204	0.05396
H	-3.29777	-2.21863	0.64764
H	-3.18937	2.06759	0.16664
H	-6.94626	-0.03095	-0.06911
C	1.83501	-0.82938	0.58770
C	2.79074	-0.31424	1.47174
C	2.25179	-1.72225	-0.41273
C	4.13695	-0.63894	1.33529
C	3.59729	-2.06374	-0.50313
C	4.55848	-1.53009	0.35684
H	2.47701	0.35953	2.27454
H	1.53051	-2.13731	-1.11398
H	5.61155	-1.79846	0.25756
C	-5.82040	2.44898	-0.25906
F	-7.08240	2.43552	0.16599
F	-5.19867	3.46910	0.33641

F	-5.84600	2.71718	-1.56632
C	-5.93787	-2.53911	0.28880
F	-5.45261	-3.37294	1.20807
F	-7.23160	-2.35090	0.55169
F	-5.85863	-3.16065	-0.88816
C	4.06079	-2.93872	-1.64053
F	5.06328	-3.73342	-1.26770
F	3.08333	-3.70254	-2.11677
F	4.51187	-2.18900	-2.65335
C	5.12306	0.05724	2.23496
F	6.34429	-0.45772	2.13482
F	5.20644	1.35743	1.92140
F	4.75122	-0.00543	3.51362
